# Perfluoroalkyl substances exposure and immunity, allergic response, infection, and asthma in children: review of epidemiologic studies

**DOI:** 10.1016/j.heliyon.2021.e08160

**Published:** 2021-10-12

**Authors:** Haley von Holst, Pratibha Nayak, Zygmunt Dembek, Stephanie Buehler, Diana Echeverria, Dawn Fallacara, Lisa John

**Affiliations:** Battelle Memorial Institute, 505 King Ave, Columbus, OH, 43201, USA

**Keywords:** PFAS, Perfluoroalkyl substances, Infant and child health, Immune function, Allergy, Infection, Asthma

## Abstract

**Background:**

Increased exposure to perfluoroalkyl substances (PFAS) potentially affects infant and childhood health through immunosuppression. Given rapidly evolving research on PFAS, it is important to comprehensively examine the impact of PFAS exposure among the pediatric population as new research becomes available due to potential fragility of the developing immune system.

**Objectives:**

This review assessed the effects of PFAS fetal, infant and childhood exposures upon the development of immune function during early life stages.

**Methods:**

Researchers completed a literature review, searching PubMed for human studies published since 2010 for PFAS and health outcomes among infants and children. Included articles incorporated key search terms in the title or abstract; non-research reports and non-English papers were excluded. The search identified 518 studies for possible inclusion. Following hands-on review, 34 were determined relevant. Subsequent analyses found 8 additional relevant articles, totaling 42 studies.

**Results:**

Major immune-related sequelae from PFAS exposures on infant and child health outcomes documented in recent literature include:

• Strong indication of immunosuppression, with diminished childhood antibody response to vaccination, particularly with PFOA, PFOS and PFHxS exposures.

• Some indication of increased risks of childhood infectious diseases/infections, particularly from PFOS exposures.

• Limited indication of an effect of PFAS exposure on allergic reactions/allergen specific IgE antibodies.

• Limited indication of an effect of PFAS exposure on atopic dermatitis (AD).

• Limited indication of an effect of PFAS exposure on asthma and lung function.

**Conclusion:**

This review summarizes recent findings of PFAS effects on infant and childhood immune health. Evidence of immunosuppression, diminished vaccine efficacy, and increased risk of infections, allergies, asthma and AD were described following *in utero*, infant, and early childhood PFAS exposures. Further investigation is warranted to characterize PFAS exposure pathways and potential modes of action in relation to PFAS effects on the developing immune system. Incontrovertible proof of PFAS immunotoxic effects could optimally be obtained by a large prospective study cohort of mothers and children from infancy through school-age. Regular assessments of circulating antibodies and response to infant and childhood vaccines during growth years could prove invaluable.

## Highlights

• This review summarizes findings of PFAS effects on infant and child immune health.
• *In utero*, infant, and early childhood PFAS exposures were examined.
• Strong evidence of PFAS exposure on diminished childhood antibody vaccination response.
• Moderate evidence of PFAS exposure on increased risk of childhood infectious diseases.
• Limited evidence of PFAS exposure on allergic reactions, atopic dermatitis, asthma.

## 1 Introduction

Early exposure to environmental agents can permanently alter developing immune systems (MacGillivray and Kollmann, 2014), and may affect immune response in infants and children. Widespread environmental per- and polyfluoroalkyl substances (PFAS), synthetic water- and oil-resistant chemicals used in multiple industrial applications and consumer products such as fire-fighting foam, Teflon coating, and food packaging are of concern (Environmental Protection Agency, 2020). Most adults have a PFAS body burden from ingestion of contaminated food (primarily fish and meats) and drinking water and PFAS-containing commercial household product use (Agency for Toxic Substances and Disease Registry, 2018; Kato et al., 2011). PFAS exposure may have adverse effects on children's health including metabolic function, neurodevelopment, and the immune system, and adversely impact pre-and postnatal development and growth (Blake and Fenton, 2020), but effects differ by type of PFAS. Conclusive evidence is needed to document the effects of these PFAS on human health. Although there are several adverse effects associated with PFAS exposure in humans, this review focuses on the pediatric population which do not have a fully developed immune system, and the potential those exposures may have on long-term health effects.

Prenatal PFAS exposure occurs through *in utero* placental transfer and postnatal exposure occurs through breastfeeding and hand-to-mouth behavior (Mogensen et al., 2015; Xue et al., 2007). PFAS in the home environment places infants and young children at increased risk of exposure due to crawling and playing behaviors. Other exposure routes in children and adults include inhalation and contaminated food and water ingestion (Agency for Toxic Substances and Disease Registry, 2020). PFAS concentrations are highest in the first 20 months of age and estimated daily intake (EDIs) relative to body weight is higher in children than adults (Winkens et al., 2017). Additionally, children with increased caloric needs may consume more than adults per pound of body weight (National Heart Lung and Blood Institute, 2013).

Early life PFAS exposures may play an important role in childhood disease susceptibility, as compromised immune system development may lead to immune dysfunction. Recent evidence suggests gestational PFOS exposure is associated with decreased vaccine-induced antibody production, indicating immunosuppression (Pachkowski et al., 2019). PFAS research focusing on children has increased substantially over the past decade. This review synthesizes recent epidemiological evidence of prenatal and child PFAS exposure and immune function effects including vaccine induced antibody response, infectious diseases, allergies, and asthma.

## 2 Methods

Researchers conducted a systematic review for this study. A search using Boolean operators and key terms related to PFAS and child health was conducted in PubMed on June 19, 2020. To ensure the capture of recently published literature, a second search was conducted on July 20, 2021. Key search terms were required to appear in the article title or abstract. Articles included in the review were published in 2010 or later, involved human research (i.e., excluded animal and toxicological findings), and included a health outcome related to children (age <18 years) and PFAS exposure. Articles were excluded if they were opinion pieces, review articles, or in a non-English language. Literature reviews were excluded to focus the analysis on primary literature. Pre-2010 literature was omitted to optimally focus on most recent PFAS research findings. The complete list of eligibility criteria can be found in Table 1.

**Table 1:** Article inclusion and exclusion criteria.

Inclusion Criteria	Exclusion Criteria
• Published 2010-present • Children/infants/neonatal age <18 years • PFAS exposure related to immune outcomes including but not limited to asthma, allergies, antibody response, and infectious diseases	• Non-English • Animal studies • Toxicology studies • Review or methodology studies that did not include unique data • Gray literature including opinion pieces, conference proceedings, dissertations, and white papers

The final July search string included (PFAS OR PFOS OR PFOA OR GenX OR PFHxS OR perfluoro∗ OR polyfluoro∗) AND (child∗ OR pregnan∗ OR maternal OR youth OR fetal OR infant OR “breast milk” OR prenatal OR postnatal) AND (immun∗ OR hypersensit∗ OR allerg∗ OR asthma OR wheez∗ OR “lung function” OR “atopic dermatitis” OR eczema OR vaccine OR antibod∗ OR autoimmun∗ OR “infectious disease” OR infection) NOT (polychromatic OR flowcytometry OR femoral OR patellofemoral OR osteoarthritis OR amputation OR “fetal alcohol syndrome” OR femur OR foramen OR trochanteric OR “personal fall” OR “fall avoidance” OR “fluorine-18” OR passport OR cryo∗ OR prefrontal OR needle OR pollen).

**Figure 1:**
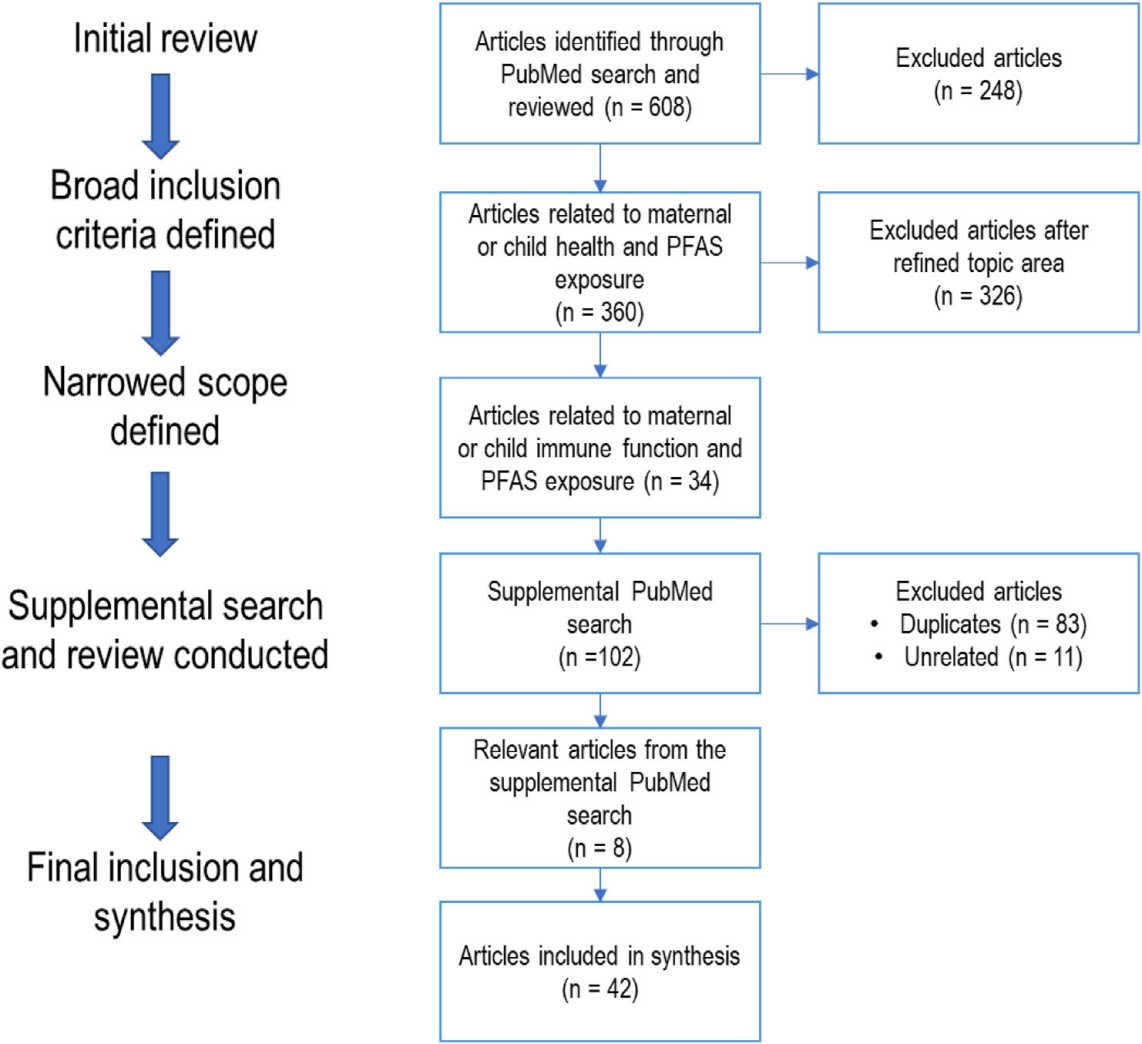
Article review flow diagram.

The initial search yielded 608 potentially relevant results (Figure 1). The search results were exported into an Excel template and three researchers reviewed the results for relevancy. The review process began with a title review to exclude unrelated results (e.g., partial fetal alcohol syndrome (PFAS)). Researchers reviewed the remaining articles by assessing the article abstract or full-text against the eligibility criteria. The three researchers met weekly to discuss relevancy and inclusion in the report. Relevant outcomes of interest included a) immunosuppression, b) hypersensitivity, and c) autoimmunity (see Table 2). Of the 360 articles, 34 were identified as relevant. The original search did not narrow the results by immune-related outcomes, that was addressed by subsequently setting inclusion criteria to capture immune-related effects for review inclusion. A supplemental updated search conducted in July 2021 yielded an additional 102 results. Of the 102 resulting papers, 83 were identified as duplicates. An additional 8 relevant articles were identified and included, resulting in a total of 42 studies included in this review.

**Table 2:** Immune related measures of health outcomes.

Immune response	Measures of immune function	Health outcomes
a) Immunosuppression	Reduced antibody production is an indication of decreased immune function or immunosuppression that may indicate a greater risk of disease.	Decreased vaccine induced antibody response, infections
b) Hypersensitivity	Exaggerated allergic responses when exposed to foreign agents.	Allergic reaction, atopic dermatitis (dermal response), asthma (respiratory response), total IgE (circulating)
c) Autoimmunity	Autoimmune disease and related effects as a result of immune responses against self-molecules.	Celiac disease, juvenile dermatomyositis, lupus

## 3 Results

**Figure 2:**
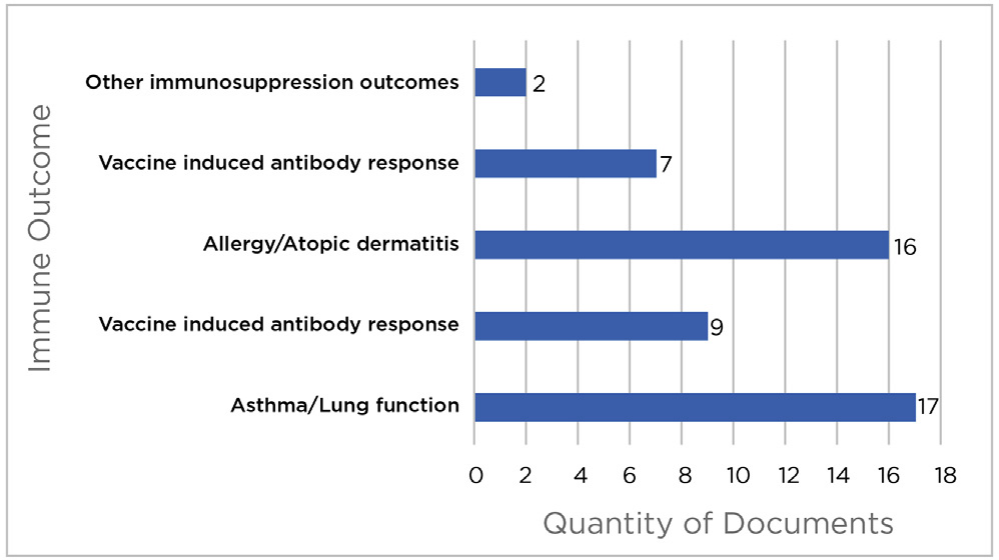
Studies included in this review across immune response outcomes.

Across the 42 included articles, two overarching immune responses, immunosuppression and hypersensitivity, were found to be the focus of studies relating PFAS exposures and child and fetal health. These studies are discussed in detail by immune outcome (Figure 2). We specifically summarize immunosuppression related to 1) vaccine induced antibody response, 2) infectious diseases and severity of symptoms of infection, and 3) other immunosuppression outcomes (e.g., hematological response markers). Hypersensitivity is discussed relevant to 4) allergic reaction, 5) atopic dermatitis (AD), and 6) asthma, lung function and biomarkers of asthma. No studies on child autoimmune diseases were captured in our review.

### 3.1 Immune suppression

Several immunosuppression health outcomes have been studied in association with PFAS exposure in children including antibody levels in response to tetanus and diphtheria (Td) and measles, mumps, and rubella (MMR) vaccines; and risk or severity of infection. Immunosuppression is initiated through a variety of mechanisms of action (MOA), the effects of which may be of greater consequence in children, as the immune system is rapidly developing and will not be fully immunocompetent until adulthood. Mechanisms of action have not been well-characterized in children; however, some associations have been described. Adverse effects on the production, maturation, and/or differentiation of T or B lymphocytes may result in reduced antibody response, and decreased CD4:CD8 ratios and NK-cell function can lead to increased risk of infection due to a wide range of pathogens (Cano and Lopera, 2013; Le Deist and Fischer, 2008).

#### 3.1.1 Vaccine induced antibody response

Several studies have attempted to understand the link between PFAS exposure and antibody production in response to diphtheria and tetanus toxoid vaccinations and MMR vaccines.

##### 3.1.1.1 Diphtheria and tetanus

**Figure 3:**
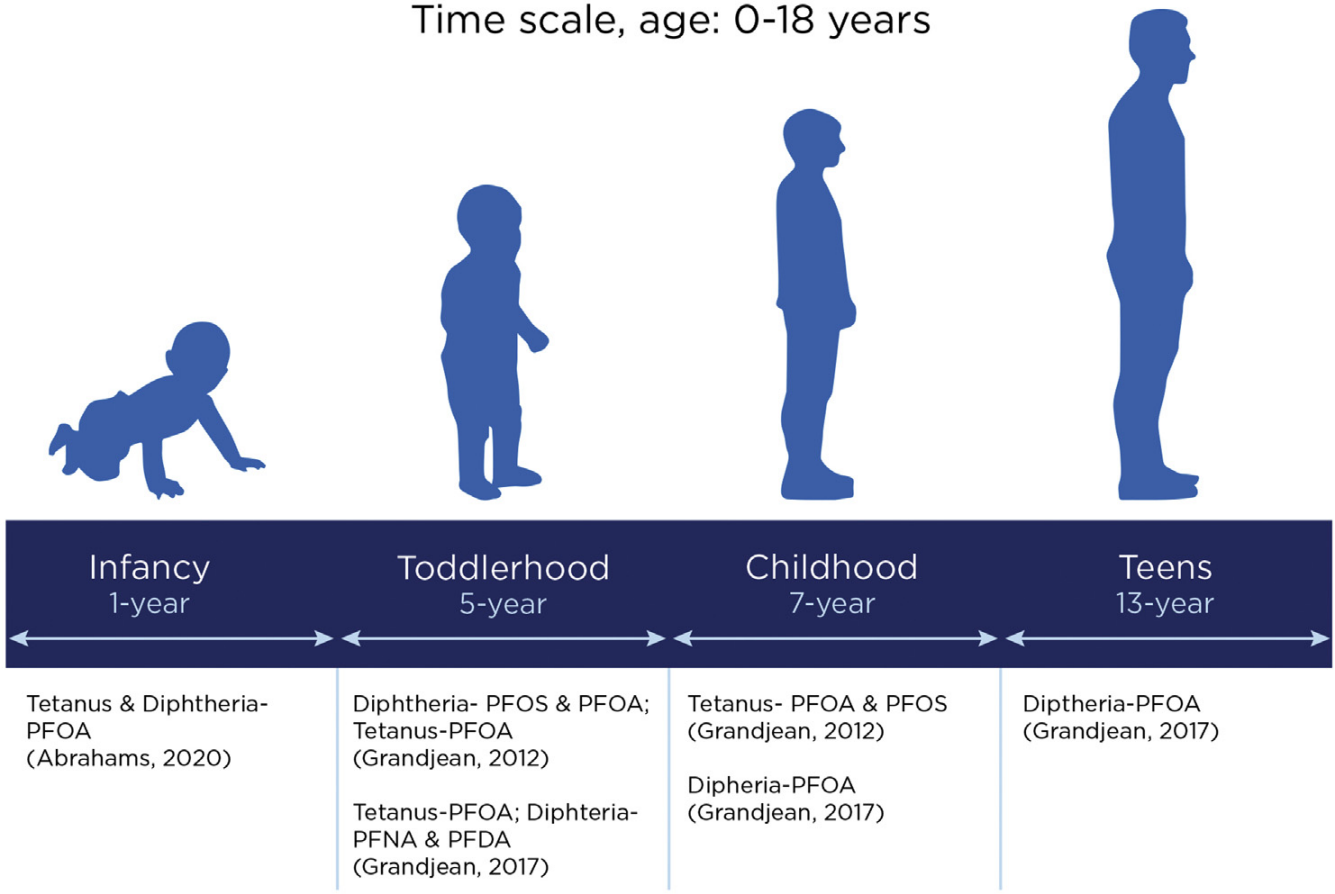
Association between type of PFAS and diphtheria and tetanus vaccine response among children between ages 1–13 years across studies.

The diphtheria, tetanus and pertussis (DTaP) vaccine is provided to children under 7 years of age, typically in five doses, at 2, 4, 6, 15–18 months, and 5 years of age (Department of Health and Human Services & Centers for Disease Control and Prevention, 2020). Studies suggest a link between maternal PFAS blood serum level and reduced antibody production following the inoculation of these vaccines in children (Grandjean et al., 2012). Epidemiological evidence on vaccine-induced antibody levels with PFAS for children aged 1 year and older suggests a decrease in tetanus and diphtheria antibodies across age groups. Among 1-year-old children a consistent inverse association between levels of vaccine antibodies for anti-Hib (IgG), anti-tetanus (IgG and IgG1) and anti-diphtheria (IgG) was found in relation to PFOA blood serum concentrations (Abraham et al., 2020). This association was also found among older children between ages 5–13 years. In Faroese birth cohort studies, childhood and infancy exposure to PFAS were associated with lower anti-vaccine antibody levels for diphtheria and tetanus in children aged 5–13 years (Grandjean et al., 2012; Grandjean et al., 2017a, Grandjean et al., 2017b; Mogensen, Grandjean, Heilmann, et al., 2015). At 5 years of age, for each doubling of PFAS exposure that occurred in early infancy, a statistically significant decrease of 19–29% in tetanus antibody concentrations were observed (Grandjean et al., 2017a, Grandjean et al., 2017b). Pre-booster antibody concentrations at 5 years of age showed the strongest negative correlations with pre- and postnatal PFOS exposures for diphtheria antibodies. At age 7, both postnatal PFOS and PFOA exposure were associated with an increased likelihood of tetanus antibody titers below the protective level (Grandjean et al., 2012). Another study noted that at 7 years of age, perfluorodecanoic acid (PFDA) serum levels were associated with decreased diphtheria antibodies (Grandjean et al., 2017a, Grandjean et al., 2017b). A study performed among 13-year-olds showed that diphtheria antibody concentration decreased with elevated serum PFAS concentration, with statistically significant decreases of ∼25% in diphtheria antibody titers for each doubling of exposure (Grandjean et al., 2017a, Grandjean et al., 2017b). These findings (Figure 3) suggest that PFAS can suppress antibody response to diphtheria and tetanus vaccination and that the impact varies by the type of PFAS.

##### 3.1.1.2 Measles, mumps and rubella

The measles, mumps and rubella (MMR) vaccine is typically administered to children in two doses: at 12–15 months and at 4–6 years of age. Two studies from a Norwegian prospective birth-cohort (BraMat), a subcohort of the Norwegian Mother and Child Cohort Study (MoBa), investigated the association between prenatal PFAS exposure (determined in maternal blood at birth) and response to MMR titer antibodies at age 3 years. Prenatal exposure to increasing levels of PFAS (PFOA, perfluorononanoic acid (PFNA), perfluorohexane sulfonate (PFHxS), and PFOS), were associated with lower antibody titers against rubella in early childhood suggesting an immunotoxic effect of PFAS (Granum et al., 2013). Gene expression analysis in cord blood from these children revealed a transcription level association between lower antibody titers and increased long-chain prenatal PFAS exposure levels (Pennings et al., 2016). A secondary analysis of data from a randomized controlled trail examined the association of PFAS exposure and antibody response to measles vaccination among children from Guinea-Bissau (Timmermann et al., 2020). Elevated serum PFAS concentrations, specifically perfluorooctane sulfonic acid (PFOS) and perfluorodecanoic acid (PFDA) were associated 21% (95% CI: 2, 37%) and 25% (95% CI: 1, 43%), respectively with reduced pre-vaccination measles antibody concentrations at the 9-month visit among the children who had received a measles vaccine. In one study, prenatal exposure to PFAS was measured using maternal blood samples collected up to 3 days after delivery, and antibody titers against rubella were measured at 3 years old. Findings showed an association between PFAS serum levels as well as rubella titers suggesting an immunotoxic effect of PFAS (Pennings et al., 2016). A second study in exposed 3-year-old children also reported lower levels of anti-rubella antibodies potentially associated with increasing levels of PFAS (PFOA, perfluorononanoic acid (PFNA), perfluorohexane sulfonate (PFHxS), and PFOS) in prenatal maternal blood (Granum et al., 2013).

In 12-19-year-old adolescents, one cross-sectional study showed exposure to higher PFAS levels was associated with lower antibody titers for mumps and rubella but not for measles (Stein et al., 2016). Among seropositive individuals, increased blood serum levels of PFOS were associated with a 5.9% decrease in mumps antibody titers and a 13.3% decrease in rubella antibody titers (Stein et al., 2016). A recent analysis of the National Health Nutrition Examination Survey (NHANES) results for children aged 12–18 years showed no association between serum rubella titers and PFOA or PFOS levels (Pilkerton et al., 2018). These findings vary by study and vaccine component, limiting the ability to draw overarching conclusions.

#### 3.1.2 Infectious diseases and symptoms of infection

Multiple articles discussed findings related to infectious diseases and PFAS exposure. Fei et al. (2010) examined maternal serum levels of PFOS and PFOA at 8 weeks gestation and the number of times children were hospitalized due to infections, using hospital discharge data from birth through 11 years of age. After controlling for numerous factors (e.g., breastfeeding duration, socioeconomic status, maternal age, etc.), the authors found no significant associations between prenatal PFOS exposure and hospitalizations from infectious diseases. Infectious diseases categorized with the most occurrences included respiratory diseases (e.g., common cold, bronchitis) and infectious (e.g., streptococcus) and parasitic diseases. Children with increased exposure to PFOA were less likely to be hospitalized. However, this was only observed when comparing the group of mothers with the lowest PFAS levels (i.e., first quartile) with those in the second quartile. Research has also found that prior to receiving a measles vaccination, children (aged 4–7 months) with higher PFAS levels had less antibodies for measles, however, results were not statistically significant (Timmermann et al., 2020). At 9 months a statistically significant relationship was found with increased levels of PFOS related to reduced measles antibodies by approximately 27% (Timmermann et al., 2020).

Two studies examined childhood symptoms of infectious disease from birth through 4 years. Dalsager et al. (2016) evaluated whether prenatal serum PFAS levels (at 10–16 weeks gestation) were associated with the occurrence (i.e., days with symptoms) of children's symptoms of infections (i.e., fever, cough, nasal discharge, diarrhea, and vomiting) from ages 1–4 years. The authors focused primarily on the presence of fever as a strong indicator of infection. After controlling for maternal age, child age, parity, and education level, a positive association was observed between prenatal exposure to PFOS and PFOA and the frequency of fever. Dalsager et al. (2021) later conducted a similar study examining prenatal PFAS levels (at 8–16 weeks gestation) and the rate of child infectious diseases resulting in hospitalization. Across types of infectious diseases, PFOS was associated with increased hospitalization rates. Further, PFOA was associated with higher risk of lower respiratory tract infections. A decrease in gastrointestinal infections was reported among higher PFAS levels.

The remaining studies looked at the presence of infection and PFAS exposure from birth through 10-years-old. Goudarzi et al. (2017) assessed maternal PFAS levels (at 28–32 weeks gestation) and childhood infectious diseases from birth through 4 years. The highest PFOS levels were associated with increased odds ratios of total infectious diseases. PFHxS was associated with a higher risk of total infectious diseases only among girls. No association was found between infectious diseases and other PFAS. Okada et al. (2012) measured the serum levels of maternal PFOS and PFOA and infectious diseases in infants from birth to 18 months. The study found no significant associations among maternal PFOS and PFOA levels and otitis media. Granum et al. (2013) examined prenatal PFAS exposure and common colds in children from birth through age 3 years. A positive association was noted between prenatal concentrations of PFOA and PFNA at the time of delivery and the number of episodes of common colds for children. Further, the authors found a positive association between prenatal PFOA and PFHxS serum concentrations and the number of gastroenteritis episodes in children from birth through 3 years old (Granum et al., 2013). Impinen et al. (2018) measured cord blood PFAS and occurrence of respiratory tract infections from birth through age 10. Among 2-year-olds, PFUnDA was positively associated with common colds. PFOS, PFOA, PFOSA, PFNA, and PFUnDA were all positively associated with lower respiratory tract infections through age 10. Impinen et al. (2019) measured maternal plasma PFAS and infectious diseases in children from birth through age 3 and between 6-7 years old. For the 0–3 age group, common colds were negatively related to PFOS and PFOA, bronchitis/pneumonia was positively associated with PFOS, PFOA, PFHxS, and PFHpS, streptococcus was positively associated with PFNA, ear infections were positively associated with PFHxS and negatively related to PFOS and PFUnDA, urinary tract infections were negatively related to PFOS, PFOA, and PFHpS, and no significant findings were found for gastric flu. For ages 6–7, bronchitis/pneumonia and ear infections had no significant associations, and gastric flu was positively associated with PFOA and PFHxS. Ait Bamai et al. (2020) also examined prenatal exposure to PFAS and doctor diagnosed infectious diseases at 7 years old. Results found an inverse association between chickenpox, otitis media, and Respiratory syncytial virus (RSV) and PFDoDA, PFTrDA, and PFOS. However, after stratifying by quantity of siblings, for children with no siblings, PFDA was positively associated with pneumonia and PFOA was positively associated with RSV. PFOA was also significantly positively associated with pneumonia when not stratifying by sibling. Lastly, Pennings et al. (2016) looked at genes associated with the common cold and the overlap of prenatal PFAS levels. Findings revealed 27 genes significantly correlated to prenatal levels of PFOS, PFOA, PFNA, or PFHxS. The authors elaborate that the gene expression related to increased PFAS levels was indicative of immunotoxicity because of association with immunomodulatory genes (e.g., CYTL1, IL27).

Overall evidence on the association between infectious diseases during childhood and PFAS exposure is limited. However, out of the studies reviewed, the preponderance of evidence suggests potential links between prenatal exposure to PFOS, PFOA, PFNA, and PFHxS and increased infectious disease occurrence later in life.

#### 3.1.3 Other immunosuppressive outcomes

Hand, foot and mouth disease (HFMD) is highly infectious and most common among children under 5 years of age. One cohort study examined prenatal PFAS exposure and HFMD antibodies among 3-month-old infants (Zeng et al., 2019). Findings suggest that prenatal PFAS exposure measured in umbilical cord blood is associated with lower HFMD antibodies in infancy (Zeng et al., 2019). A doubling in the composite sum of PFAS was associated with significant increase in the risk of HFMD antibody concentration below clinical protection levels for Coxsackie A16 (CA16) and Enterovirus 71 (EV71) viruses, common causes of HFMD.

### 3.2 Hypersensitivity

Human exposure to environmental agents can provoke a hypersensitive reaction mediated by immunological mechanisms resulting in a range of allergic reactions (Muñoz-Carrillo et al., 2017). This hypersensitivity reaction can be antibody mediated or cell mediated and typically affects the skin, gut, and respiratory tract (Muñoz-Carrillo et al., 2017). Summaries of studies on hypersensitivity including allergic reactions, dermal effect such as AD and respiratory response including asthma are presented below.

#### 3.2.1 Allergy serum biomarkers

Some PFAS compromise the immune system during pre-and postnatal periods (Rappazzo et al., 2017), which may contribute to the etiology of allergic conditions. Okada et al. (2012) measured the serum levels of maternal PFOS and PFOA from 2002-2005 to study the association with infant allergies and infectious diseases from birth to 18 months. Concentrations of umbilical cord serum IgE measured at birth were significantly decreased in association with high maternal PFOA concentrations among female infants. The underlying mechanism for observed elevations of IgE related to PFAS exposures is not fully understood and the literature reflects conflicting results. A study of the Taiwan Birth Panel cohort examined the association between cord blood serum PFAS level (serum concentrations of PFOA, PFOS, PFNA, and PFHxS), cord blood IgE levels, and AD in 2 year-olds (Wang et al., 2011). Of the 244 study participants, 17.6% developed AD. The authors report no significant associations between PFAS and AD. However, prenatal PFOA and PFOS were associated with increased cord blood IgE levels, especially in boys (Wang et al., 2011). In contrast, a trans-Canada cohort study of the Maternal-Infant Research on Environmental Chemicals (MIREC) found that PFAS were not associated with immunotoxic effects and no statistically significant non-linear associations were observed for IL-33/TSLP or IgE (Ashley-Martin et al., 2015).

Beyond IgE, minimal studies examined other biomarkers of allergens such glutathione S-transferase genotype and basophil counts. One study conducted by Oulhote et al. (2017) found high basophil counts were associated with increased PFAS levels in 5-year-olds. Although the study didn't examine allergies, basophil counts have been found to correlate with serum total IgE levels (Dong et al., 2013). A cohort study examined the effect of PFAS exposure and glutathione S-transferase (GST) genotype interaction on AD among Taiwanese children (Wen et al., 2019). *In utero* PFOA exposure among infants with GST null genotypes was also associated with increased risk of developing AD (Wen et al., 2019).

#### 3.2.2 Clinical allergic diseases

Okada et al. (2012) also measured maternal PFOS and PFOA and associated allergies among infants from birth to 18 months. The study found no significant associations among maternal PFOS and PFOA levels and food allergy, eczema, or wheezing in 18-month-old infants (Okada et al., 2012). A second study by Okada et al. (2014) measured maternal plasma to examine the role of long-chain PFAS and its effects on infant allergies at 12 and 24 months. Associations between high maternal PFAS levels including PFOA, PFNA, perfluoroundecanoic acid (PFUnDA), perfluorododecanoic acid (PFDoDA), and perfluorotridecanoic acid (PFTrDA) and a decline in risk for developing at least one of the following: eczema, wheezing, and allergic rhinoconjunctivitis symptoms among female infants were found. Furthermore, prenatal exposure to PFTrDA was associated with a decreased risk of eczema development in female infants. However, among male infants, the risk of allergic disease was not associated with maternal PFAS levels. PFAS association with eczema was consistent when children were followed up through 4 years of age. Another study examined prenatal exposure to PFAS and its relation to childhood allergies diseases until the age of 7 years. It was observed that prenatal exposure to PFAS (PFOA, PFUnDA, PFDoDA, PFTrDA, and PFOS) was associated with reduced risks of childhood eczema (Ait Bamai et al., 2020). A study conducted among 1,024 mother–child pairs from Greenland and Ukraine found limited evidence to support a link between prenatal exposures to PFASs with eczema symptoms in 5- to 9-year-old children from INUENDO birth cohort (Smit et al., 2015).

Goudarzi et al. (2016) found that PFHxS, PFNA, PFUnDA, and PFDoDA are also negatively associated with the prevalence of child allergic outcomes after prenatal exposure. The study found that exposure to PFDoDA and PFTrDA was associated with reduced risk of eczema in 4-year-old children. Additionally, exposure to PFHxS was negatively associated with wheezing prevalence. Allergic symptoms and PFDoDA or PFTrDA exposure were found to be statistically significant only in boys. Compared to the previous study which included sex as a confounder in adjusted models, this study stratified analysis by sex, which may have led to differences in findings across these studies. In summary, prenatal exposure to long-chain PFAS, such as PFDoDa and PFTrDA, may have an immunosuppressive effect and may disrupt immune system balance among 4-year-old children (Goudarzi et al., 2016).

A few studies have examined the association between PFAS and AD. Wen et al., 2019a, Wen et al., 2019b investigated the association between prenatal PFOA and PFOS exposure, as measured in cord blood, and early onset of AD in children (3–60 months old). The results indicate *in utero* exposure to PFOA was associated with higher risk of earlier AD in children <5 years of age. No association was found between *in utero* PFOS exposure and AD (Wen et al., 2019). Similar findings were noted in a prospective birth cohort study that examined PFAS umbilical serum levels and childhood AD between 6-24 months of age (Chen et al., 2018). Among 687 children who completed a 24-month follow-up visit, 25.2% developed AD. PFOA concentration in cord blood was significantly associated with a 2.1-fold increase in AD risk. When stratified by gender, prenatal exposures to PFOA, PFDA, perfluorododecanoic acid (PFDoA) and PFHxS were significantly associated with childhood AD in girls during the first 24 months. The biological mechanism for this sex difference is unclear (Zhou et al., 2016). These studies provide evidence for the potential effect of PFAS on children's immune systems (Chen et al., 2018).

#### 3.2.3 Asthma, lung function, and biomarkers of asthma

Understanding the relationship between PFAS and asthma may contribute to knowledge on the effect of PFAS on the immune system. According to Lloyd and Hessel (2010), asthma may be related to the immune system through the contribution of type-2 T helper cells, and potentially other T cells. Further, inflammation is a common immune response to environmental agents such as allergens. Asthma is considered a hypersensitive immune response because it is thought to be over-responding to a factor that may not necessitate that response. Numerous studies were identified as related to PFAS exposure and asthma, lung function or biomarkers of asthma in children. Asthma assessment differed significantly across studies, with some tracking asthma diagnosis from a physician or lung function tests (e.g., forced expiratory volume or forced expiratory flow) and others measuring asthma biomarkers like urine CC16.

##### 3.2.3.1 Asthma

In children ages 3–11 years, Jackson-Browne et al. (2020) found no overall association between asthma and PFOS, PFOA, PFHxS, and PFNA exposure. However, an interaction with age was identified. Specifically, there was a 70% increased risk of asthma for each standard deviation increase in PFOS levels among children ages 3–5 years versus a 10% increase among children ages 6–11 years. A lack of significant findings among childhood asthma diagnosis and/or symptoms (e.g., wheeze) and PFAS levels has been reported by numerous other studies (Impinen et al., 2018, 2019; Jackson-Browne et al., 2020; Smit et al., 2015). For example, Beck et al. (2019) examined prenatal exposure to PFOS, PFOA, PFHxS, PFNA, and PFDA and child asthma at the age of 5 years. No significant relationships were found for doctor-diagnosed asthma and prenatal PFAS levels. Some evidence has even found increased PFAS exposure is related to decreased risk of asthma. Manzano-Salgado et al. (2019) reported a negative association between asthma risk and prenatal PFNA exposure among children through the age of 7 years. Using NHANES data from slightly older adolescents (ages 12–19 years), Humblet et al. (2014) found a positive association between PFOA and childhood asthma diagnosis, and an inverse association for PFOS levels and asthma and wheezing. Qin et al. (2017) measured PFAS exposure among children diagnosed with asthma (*n* = 132; ages 10–16), and with no diagnosis of asthma (*n* = 168; ages 10–16). Across both groups (*N* = 300), asthma presence was positively associated with PFOS, PFOA, PFHxS, PFNA, and perfluorotetradecanoic acid (PFTA). No significant associations were identified for perfluorobutane sulfonic acid (PFBS), perfluorohexanoic acid (PFHxA), and PFDA after adjusting for variables such as body mass index, age, and environmental tobacco smoke exposure. Looking at a similar age group, Kvalem et al. (2020) examined the effects of child PFAS exposure on asthma among 10- and 16-year-olds. PFHpA was associated with increased risk of asthma among 10-year-old girls. However, no other significant relationships were identified for PFOA, PFNA, PFDA, PFUnDA, PFHxS, PFHpS, PFOS, and PFOSA. In a similar age group (ages 10–15 years), Dong et al. (2013) found children with increased exposure to PFOS, PFOA, PFBS, PFDA, PFDoA, PFHxS, and PFNA had an increased risk of asthma. Similarly, Averina et al. (2019) studied older adolescents PFAS levels and risk of asthma. Adolescent asthma and blood samples were measured at around age 16 and 18 years. Asthma was measured by self-reported doctor diagnosed asthma. At the age of ∼16 years, PFOS and PFHxS were positively associated with asthma whereas at ∼18 years, only PFOS was positively associated with asthma. In a study conducted by Zhou et al., 2017a, Zhou et al., 2017b results found children with asthma had significantly higher levels of all PFAS evaluated in the study including PFOA, PFDA, PFHxS, and PFNA.

A study by Timmermann et al. (2017) examined the relationship between PFAS exposure and asthma as moderated by the MMR vaccine. Both pre-natal and child PFAS levels were assessed. The authors found increased PFAS exposure among children at 5 years old was sometimes related to higher asthma incidence among unvaccinated children but not vaccinated children. The authors summarize, “while PFAS exposure may impact immune system functions, this study suggests that MMR vaccination might be a potential effect-modifier” (p. 2).

##### 3.2.3.2 Lung function

Qin et al. (2017) also examined lung function and child PFAS exposure. Using electronic spirometers numerous measures of lung function were examined, including forced expiratory volume in 1 s (FEV), forced vital capacity (FVC), peak expiratory flow rate (PEF), and forced expiratory flow (FEF). Multivariate linear regression was done for each independent measure of lung function (FEV, FVC, PEF, FEF) among children with asthma. Four PFAS were significantly associated with multiple measures of lung function. Specifically, higher PFOS and PFNA levels were significantly associated with lower FVC and FEV; higher PFOA levels were significantly associated with lower FEV and FEF; and higher PFHxS levels were significantly associated with lower FVC and FEV. No significant results were found for PFBS, PFDA, PFHxA, and PFTA. Similarly, Agier et al. (2019) examined prenatal exposure to PFNA and PFOA and subsequent child FEV1%. Lung function was measured between ages 6–12 years in children from the European Human Early-Life Exposome (HELIX) cohort. Significantly lower child FEV1% associated with higher PFNA and PFOA maternal serum. However, these results were not significant after correcting for multiple testing.

Gaylord et al. (2019) assessed PFAS exposure and lung function among children near the World Trade Center on September 11, 2001 compared to a control group. The children enrolled in the World Trade Center Health Registry (WTCHR) were previously found to have higher levels of PFHxS, PFOS, PFOA, PFNA, and PFDA compared to a matched control group. Lung function and PFAS levels were measured from 2014-2016. During this time, all participants were between the ages of 13–22 years. No statistically significant relationship between PFAS exposure and respiratory outcomes (5–20 Hz resistance frequency, FVC, FEV, FEV/FVC, total lung capacity, residual volume, functional residual capacity, eosinophil count, and asthma diagnosis after September 11, 2001) was found. Based on these findings, the authors suggest that PFAS exposure does not adversely affect respiratory function in children of this age group; however, the children's mean PFAS levels in the highly exposed condition were lower than anticipated.

##### 3.2.3.3 Asthma biomarkers

The biomarkers suspected to be most indicative of asthma are related to inflammation (type 2) and are attributed to T_H_2-cytokines, IL-4, IL-5, and IL-13 (Wan and Woodruff, 2016). For example, the cytokine IL-4 has been found to induce secretion of IgE which is linked to increased symptoms of asthma (Steinke and Borish, 2001). Similarly, T_H_2-cytokines lead to increased IL-5 production which can lead to eosinophilia (Greenfeder et al., 2001). Some of these biomarkers have been studied in relation to PFAS exposure and asthma among children.

Zhu et al. (2016) examined the relationship between lymphocyte function and PFAS exposure among children diagnosed with asthma (*n* = 231; ages 9–16 years), and with no diagnosis of asthma (*n* = 225; ages 11–15 years). The results found asthma in males to be statistically significantly associated with higher child serum levels of PFOS, PFOA, PFBS, PFDA, PFHxS, and PFNA. Comparatively, asthma in females was statistically significantly related to higher levels of PFOA, PFDA, and PFHxS. Further, among males with asthma, lower levels of T_H_1 cytokines were statistically significantly associated with higher levels of PFDA (IL-2 only), and higher levels of T_H_2 cytokines were statistically significantly associated with higher levels of PFOA (IL-4 and IL-5) and PFBS (IL-5 only). Among females with asthma, PFDA was statistically significantly associated decreased with T_H_1 (IFN-γ only). The authors concluded that PFAS exposure may contribute to asthma due to effects of T_H_ cell dysregulation (Zhu et al., 2016).

Dong et al. (2013) studied PFAS exposure in children ages 10–15 years with and without asthma. Serum levels among children with asthma for PFOS and PFOA were positively associated with IgE, absolute eosinophil counts (AEC), and eosinophilic cationic protein (ECP). Among children with asthma, PFHxA serum levels was the only PFAS not significantly associated to any of the biomarkers measured (IgE, AEC, and ECP). In children without asthma, no significant associations were found between the three biomarkers and PFOS, PFOA, PFBS, PFHxA, PFHxS, PFNA, and PFTA. However, ECP was positively associated with PFDA and PFDoA levels in non-asthmatic children.

Another study also conducted a case-control study that measured PFAS exposure and urine 16-kDa club cell secretoryprotein (Clara) (CC16) levels among children ages 10–15 years with asthma. CC16 levels are a biomarker for asthma in youth (Zhou et al., 2017a, Zhou et al., 2017b). PFOS, PFOA, PFBS, PFDA, PFDoA, PFHxA, PFHxS, PFNA, and PFA were measured in the serum of children with and without asthma. The researchers reported decreased CC16 levels were associated with increased PFOS, PFOA, PFBS, and PFHxA levels among children with asthma. PFAS levels were inversely associated with CC16 levels among children diagnosed with asthma, but not among children who were not diagnosed with asthma, with stronger associations found among males compared to females. Research has also examined the interaction of PFAS exposure, reproductive hormones (i.e., estradiol, testosterone) and asthma (Zhou et al., 2017a, Zhou et al., 2017b). Children with asthma had significantly higher levels of all PFAS evaluated in the study. Among children with asthma, testosterone levels were significantly negatively related to PFOS and PFOA, and levels of estradiol were significantly positively related to PFOS, PFOA, PFDA, and PFNA. Due to minimal significant findings among children without asthma, the researchers concluded that reproductive hormones may enhance the relationship between PFAS and asthma.

## 4 Discussion

Across the literature on PFAS exposure and child immune function, there is probable evidence of immunosuppressive effects, especially related to reduced vaccine response and some indication of increased risk of infectious disease or symptoms of infection. Research on hypersensitivity outcomes including asthma, allergic reaction and AD remains to be clarified. Future research should focus on more consistent research methodology in how health outcomes are measured, especially for infectious diseases (see section 4.1.2. Infectious diseases and symptoms of infection). Research also remains limited among the pediatric population; studies across children's ages with numerous temporal PFAS measurements or at known periods of susceptibility are needed. Studies with retrospective temporal analysis of PFAS deposition that offer a better documented record of PFAS exposures (discrete or multiple) would also prove beneficial for assessing childhood deficits. Some studies suggest PFAS exposure alters immune function differently by gender and this should be further explored.

### 4.1 Immunosuppression

#### 4.1.1 Vaccine antibody response

Collectively, across age groups, there was strong evidence for PFAS's association with suppression of antibody response to vaccination. The studies in this review focused on pediatric vaccination including TDaP (Tetanus, Diphtheria, Pertussis) and MMR. As described previously, elevated PFAS were associated with reduced humoral immune response to routine childhood immunizations, including lower levels of tetanus and anti-diphtheria antibodies (Abraham et al., 2020; Grandjean et al., 2012; Mogensen, Grandjean, Heilmann, et al., 2015) and for mumps (Stein et al., 2016) and rubella (Granum et al., 2013; Pilkerton et al., 2018; Stein et al., 2016) antibody titers.

PFAS exposure assessments were limited to two or three discrete time points with varied time intervals across studies. These may not fully capture entire childhood exposure and serial serum PFAS analyses might provide stronger evidence for PFAS immunotoxicity. Vaccine response studies varied in time between last vaccination and time of blood collection. Further, antibody concentration assessed at a particular time may not accurately represent immune protection level against long-term diseases.

Only two studies accounted for co-exposure to PCBs and vaccine response (Grandjean et al., 2012; Grandjean et al., 2017a, Grandjean et al., 2017b). Research on vaccine response should consider multiple exposures. For example, research demonstrates that dichlorodiphenyldichloroethylene (DDE) reduces TB antibodies, and there is a possible additive relationship with PCBs (Jusko et al., 2016). More research is needed on the additive effect of exposure to PFAS, DDE, and PCBs on child antibodies. It is important to note that one particular research group has published multiple papers based on the same cohort, that may influence subsequent inferences made (Grandjean et al., 2012; Grandjean et al., 2017a, Grandjean et al., 2017b).

#### 4.1.2 Infectious diseases and symptoms of infection

Occurrence of infectious diseases may indicate a compromised immune system. For example, impaired humoral immunity such as B cell deficiency results in increased respiratory infections (Smith and Cunningham-Rundles, 2019). Infectious diseases among children with prior PFAS exposure may indicate immune suppression via a yet undescribed MOA. However, evidence on the relationship between infectious diseases during childhood and PFAS exposure is inconsistent and limited. Emerging evidence may suggest that increased PFOS exposure is related to increased risk of infectious diseases and/or symptoms of infection among children (Dalsager et al., 2016; Goudarzi et al., 2017). PFOS was shown to increase lower respiratory tract infections, days with a fever, and total quantity of infectious diseases in children.

Of those studies measuring infectious diseases, four focused on children under 4 years of age, while three examined children from birth up to age 11. Studies should continue to assess findings among children at different ages. For example, children may have peak levels of PFOA and PFOS before 2 years of age due to increased breastfeeding and exposure through dust ingestion (Winkens et al., 2017). Further, as the immune system changes with age, genetics may play a lesser role as the child ages (Brodin et al., 2015). Thus, differentiating immune effects as PFAS levels change throughout childhood development is important.

Attempting to define the role of immune function by measuring occurrence of infection, length of symptoms, and hospitalization need consider controlling for known confounders and effect modifiers that may contribute to infectious disease susceptibility. Most relevant studies examined controlled for breastfeeding, age, SES and sex (Dalsager et al., 2016; Dalsager et al., 2021; Fei et al., 2010) and family history was not controlled in any studies examining infectious disease outcomes (Table 4). Other factors minimally explored in this literature are the roles of prenatal stress, BMI, and nutrition on immune development (Table 4). Research on immune function and risk of infections have been studied in relation to BMI, family history and stress. For example, research suggests obese children and malnourished children are at higher risks for respiratory tract infections (Dobner and Kaser, 2018). Nielsen et al. (2011) found, “Compared with nonexposed children, children exposed prenatally to stress had a 25% and a 31% increased risk of SID [severe infectious disease] or LSID [less severe infectious disease] hospitalization in childhood,” especially in children up to 1 year. Further, humoral immunity is known to be influenced by genetics making family history a potentially important, but difficult to quantify, variable (Scepanovic et al., 2018). According to Chandra (2002), malnutrition is a strong contributor to the course of infection and outcome for some infections but not others. Chandra notes that nutrition deficiency influences the course of infection strongly for pneumonia, moderately for influenza, and minimally for tetanus. Research on PFAS and infectious diseases should therefore consider nutritional deficiency for infections that are strongly influenced by diet. This would allow for better control of confounding variables, such as nutrition, should it be relevant. Further, animal studies have demonstrated that prenatal malnutrition adversely influences thymus and lymphocyte function (Pai et al., 2018; Palmer, 2011). Yet, populations where endemic malnutrition may be a problem, have not been extensively studied. No studies discussed the role that prenatal nor child diet may play in immune function and infectious diseases. Studies controlled solely for breastfeeding and thus do not comprehensively examine nutrition since they did not capture the prenatal period nor the food children consumed beyond breastmilk. To understand if prenatal exposure to PFAS affects child immune response to infectious diseases, research designs could be strengthened by assessing the need for statistically adjusting for factors like family history, BMI, prenatal stress, and nutrition to more fully capture complex influences on the immune system.

### 4.2 Hypersensitivity

#### 4.2.1 Allergy serum biomarkers

The prevalence of allergies is on the rise and approximately 18% of children suffer from at least some form of allergies (Blue Cross Blue Shield, 2018). PFAS have been reported to exhibit immunomodulatory effects among children, yet the association between PFAS and allergic disease is unclear. Numerous studies examined PFAS exposure, IgE levels, and allergy outcomes. However, only one study found cord serum IgE level among children was positively associated with prenatal exposure to PFOA and PFOS levels (Wang et al., 2011). Beyond IgE, minimal studies examined other biomarkers of allergens such as IL-33 and glutathione S-transferase genotype.

#### 4.2.2 Clinical allergic diseases

A study by Okada et al. (2014) found an association between high maternal PFAS levels and decline in risk for eczema among female infants at 12–24 months. Similarly, another study that followed infants until 4 years of age found that exposure to long-chain PFAS, including PFHxS, PFNA, PFUnDA, PFDoDA, were all negatively associated with the prevalence of allergic outcomes (Goudarzi et al., 2016). In this study the PFAS association with allergies was significant among males and not females. Some of the conflicting results observed in these studies show outcomes that differ by sex with only males or females as responders. Although research has begun to show a relationship between PFAS exposure and decreased allergic reaction, the evidence is too limited to draw conclusions. Future larger sample size studies are warranted to investigate the relationship between maternal PFAS exposure and child allergies and infections from infancy to school-age and the immunotoxin potential of PFAS.

AD is a common chronic skin inflammation disorder in children and a significant predictor of the sensitization to other allergens and respiratory allergic disease such as asthma (Somanunt et al., 2017). The biological mechanism of PFAS exposure on AD remains unclear. In Taiwan, PFOA and PFOS were associated with increased cord blood IgE levels, especially in boys (Wang et al., 2011). PFAS may cross placental barriers and enter fetal circulation or be transferred through breast-feeding during the postnatal period, resulting in altered immune responses such as the development of atopy. PFAS might augment the hypersensitive response to allergens (Fairley et al., 2007). Some studies found *in utero* exposure to PFOA associated with higher risk of earlier AD in children (Chen et al., 2018; Wen et al., 2019). Given the limited number of studies and the variability in the responses, results are inconclusive. Thus, observations made from a large number of children could prove useful. Furthermore, cord blood used to measure PFAS levels among mothers may not be an accurate representation of PFAS during their entire pregnancy (Kato et al., 2014).

#### 4.2.3 Asthma, lung function and biomarkers of asthma

Extensive research has been undertaken to understand potential environmental triggers and causes of asthma. Rivera Rivera et al. (2020) researched prenatal exposure to particulate matter and found increased exposure associated with increased presence of wheezing among 6-8-year-old children. The period of gestation was particularly sensitive beginning at 14 weeks through post gestation at the age of 18 months. Effects on T_H_2 differentiation, T_H_2 cytokine production and increased levels of IgG1 and IgE are possibly indicative of allergic asthma (Bornehag and Nanberg, 2010) and were altered in association with PFAS exposure (Zhu et al., 2016). However, thus far research on the effects of PFAS exposure on asthma development has had mixed findings. Numerous researchers have found insufficient evidence for an association between PFAS exposure and asthma (EFSA Panel on Contaminants in the Food Chain et al., 2020; European Food Safety Authority, 2020), despite results showing that nearly all serum levels of children with asthma were higher than for those without asthma (see Table 3). PFAS have been found to alter MOA related to asthma onset, and continued research is needed.

**Table 3:** Average PFAS serum levels across studies.

Citation	Population Subgroup	PFOS (ng/mL)	PFOA (ng/mL)	PFNA (ng/mL)	PFDA (ng/mL)	PFUA (ng/mL)	PFBS (ng/mL)	PFDoA (ng/mL)	PFHxS (ng/mL)	SumPFAS (ng/mL)
Immune Outcome: Allergies										
Averina et al. (2019), The Tromso study Fit Futures,	Girls (n = 335) Mean (IQR)	5.8 (2.7) sum 3.1 (1.6) lin	2.1 (1.2)	N/A	N/A	N/A	N/A	N/A	0.82 (0.56) sum 0.62 (0.48) lin	10.7 (4.9)
	Boys (n = 320) Mean (IQR)	6.8 (3.0) sum 3.5 (1.7) lin	1.9 (0.7)	N/A	N/A	N/A	N/A	N/A	0.94 (0.61) sum 0.76 (0.51) lin	11.2 (4.9)
Ait Bamai et al., 2020, Hokkaido cohort, Maternal serum at 28 and 32 weeks gestation, Japan 2003–2012 Age 0–7 years	(N = 2,206) Median	5.12	1.94	1.14	0.51	N/A	N/A	N/A	N/A	N/A
Chen et al. (2018), Birth cohort, cord blood, Shanghai 2012–2015 Age 6, 12, and 24 months	All (N = 687) Mean ± SD	2.93 ±3.11	7.73 ±3.98	0.70 ±0.29	0.45 ±0.42	0.46 ±0.34	0.05 ±0.03	0.10 ±0.07	0.18 ±0.08	N/A
	w/o AD (n = 514) Descriptive statistics not stated	2.46 (1.80–3.17)	6.76 (4.84–9.29)	0.64 (0.49–0.81)	0.36 (0.23–0.52)	0.40 (0.29–0.52)	0.05 (0.04–0.06)	0.09 (0.07–0.13)	0.16 (0.13–0.20)	N/A
	w/AD (n = 173) Descriptive statistics not stated	2.54 (1.83–3.37)	7.17 (5.22–10.19)	0.66 (0.54–0.86)	0.39 (0.26–0.58)	0.41 (0.30–0.57)	0.05 (0.04–0.06)	0.09 (0.07–0.12)	0.16 (0.14–0.21)	N/A
Wen et al. (2019), Cord blood, Taiwan 2001–2005 Age 5 years	All (N = 863) Mean ± SD	4.27 ±5.58	1.22 ±1.38	N/A	N/A	N/A	N/A	N/A	N/A	N/A
Goudarzi et al. (2016), Maternal samples, 28–32 weeks gestation, Hokkaido, Japan 2003–2013 Age 4 years	All (N = 1,558) Mean	5.46	2.71	1.40	0.58	N/A	N/A	N/A	0.32	N/A
Okada et al. (2012), Prospective cohort, cord blood after second trimester, Sapporo, Japan 2002–2005 Age 18 months	All (N = 343) Mean	5.60	1.40	N/A	N/A	N/A	N/A	N/A	N/A	N/A
Wen et al. (2019), Birth cohort, cord blood, Taipei, Taiwan, 2001–2005 Age 6 months–2 years	All (N = 839) Mean (SD)	4.24 (5.54)	1.19 (1.18)	3.27 (7.01)	N/A	N/A	N/A	N/A	25.21 (11.55)	N/A
	w/AD (n = 45) Arithmetic mean ± SE	4.71 ±0.48	1.60 ±0.18	3.07 ±0.96	N/A	N/A	N/A	0.27 ±0.05	26.99 ±1.72	N/A
	w/o AD (n = 794) Arithmetic mean ± SE	3.99 ±0.12	1.17 ±0.04	3.14 ±0.23	N/A	N/A	N/A	0.35 ±0.01	25.11 ±0.41	N/A
Wang et al. (2011), Birth cohort, cord blood, Taiwan 2004 Age 2 years	All (N = 244) Median (Range)	5.5 (0.11–48.36)	1.71 (0.75–17.40)	2.3 (0.38–63.87)	N/A	N/A	N/A	N/A	0.035 (0.035–0.420)	N/A
Ashley-Martin et al. (2015), Maternal Infant Research on Environmental Chemicals Study (MIREC), cord blood, Canada 2008–2011 Age N/A	IL-33/TSLP (pg/mL) Z ≥ 80%; <80% (n = 1,242) Geometric mean (SD)	4.7 (1.9); 4.5 (1.8)	1.7 (1.9); 1.7 (1.8)	N/A	N/A	N/A	N/A	N/A	1.0 (2.2); 1.0 (2.3)	N/A
	IgE (ku/L) ≥0.5 ku/L; <0.5 (n = 1,242) Geometric mean (SD)	4.6 ± 1.9; 4.6 ± 1.8	1.7 ± 1.9; 1.6 ± 1.8	N/A	N/A	N/A	N/A	N/A	1.0 ± 2.4; 1.0 ± 2.3	N/A
Okada et al. (2014), Hokkaido Study on Environment and Children's Health, maternal serum at 28–32 weeks gestation, Hokkaido 2003–2009 Age 12–24 months	All (N = 2,062) Geometric mean	5.01	2.08	1.19	0.501	N/A	N/A	N/A	0.275	N/A
Oulhote et al. (2017), Faroe Island Cohort, cord blood and maternal serum 2 weeks after parturition, child serum at 18 months and 5 years, Faroe Islands, 2007–2009 Age 18 months and 5 years	Maternal (n = 55) Geometric mean ± GSE	9.075 ±0.495	1.47 ±0.100	0.789 ±0.056	0.298 ±0.020	N/A	N/A	N/A	0.24 ±0.022	N/A
	18 months old (n = 41) Geometric mean ± GSE	8.25 ±0.761	3.58 ±0.391	1.244 ±0.135	0.347 ±0.033	N/A	N/A	N/A	0.247 ±0.045	N/A
	5 years old (n = 53) Geometric mean ± GSE	5.058 ±0.261	2.596 ±0.128	1.358 ±0.095	0.357 ±0.024	N/A	N/A	N/A	0.372 ±0.032	N/A
Knudsen et al., 2018, Greenlandic Birth cohort, maternal serum, Greenland 2010–2011 Age N/A	All (N = 189) Median (Range)	N/A	N/A	N/A	N/A	N/A	N/A	N/A	N/A	18.5 (6.14–96.43)
Immune Outcome: Asthma										
Agier et al. (2019), European Human Early-Life Exposome (HELIX), maternal and child serum, Europe 2003–2009 Age 6–12 years	Prenatal (N = 1,033 child-mother pairs) Mean (SD)	8.2 (6.1)	2.6 (1.8)	0.8 (0.6)	N/A	N/A	N/A	N/A	0.8 (1)	N/A
	Postnatal (N = 1,033 child-mother pairs) Mean (SD)	2.6 (2.6)	1.7 (0.7)	0.7 (0.7)	N/A	N/A	N/A	N/A	0.5 (1)	N/A
Beck et al. (2019), Odense Child Cohort, maternal serum, Denmark 2010–2012 Age 5 years	(N = 981) child-mother pairs Median (25th–75th percentile)	7.73 (5.68–10.44)	1.68 (1.13–2.35)	0.65 (0.49–0.86)	0.29 (0.22–0.40)	N/A	N/A	N/A	0.36 (0.24–0.50)	N/A
Dong et al. (2013), Genetic and Biomarkers study for Childhood Asthma, child serum, Taiwan 2009–2010 Age 10–15 years	w/o asthma (n = 225) Mean (SD)	33.4 ±26.4	1.0 ±1.1	0.9 ±0.3	1.0 ±0.5	N/A	0.5 ±0.2	4.5 ±6.0	2.1 ±2.2	N/A
	w/asthma (n = 231) Mean (SD)	45.5 ±37.3	1.5 ±1.3	1.1 ±0.5	1.2 ± 0.5	N/A	0.5 ±0.2	5.8 ±6.0	3.9 ±9.0	N/A
Gaylord et al. (2019), World Trade Center Health Registry (WTCHR) and comparison group, child serum, United States 2014–2016 Age 13–22 years	Comparison group (n = 165) Mean (SD)	3.45 (3.30)	1.53 (0.65)	0.56 (0.30)	0.13 (0.13)	N/A	N/A	N/A	0.73 (0.75)	6.5 (4.0)
	WTCHR (n = 118) Mean (SD)	4.26 (2.51)	1.91 (0.80)	0.67 (0.30)	0.17 (0.12)	N/A	N/A	N/A	1.28 (2.0)	8.44 (4.39)
Jackson-Browne et al. (2020), National Health and Nutrition Examination Survey (NHANES), child serum, United States 2013–2014 Age 3–11 years	All (N = 607) Geometric mean (IQR)	3.7 (2.6–5.5)	1.9 (1.4–2.7)	0.7 (0.5–1.1)	N/A	N/A	N/A	N/A	0.8 (0.5–1.3)	N/A
	w/o asthma (n = 504) Geometric mean (IQR)	3.7 (2.5–5.4)	1.9 (1.4–2.7)	0.8 (0.5–1.3)	N/A	N/A	N/A	N/A	0.8 (0.5–1.2)	N/A
	w/asthma (n = 93) Geometric mean (IQR)	4.0 (2.9–5.6)	2.0 (1.4–2.6)	0.8 (0.5–1.3)	N/A	N/A	N/A	N/A	0.9 (0.6–1.4)	N/A
Kvalem et al. (2020), Environment and Child Asthma (ECA) Study, Norway 1992–1993 Age 2, 10, and 16 years	All (N = 378) Mean (SD)	20.9 ±8.75	4.62 ±1.86	0.63 ±0.30	0.19 ±0.11	N/A	N/A	N/A	3.33 ±9.62	N/A
Manzano-Salgado et al. (2019), Spanish INMA birth cohort study, maternal plasma, Spain 2003–2008 Age 0–7 years	All (N = 1,243) Mean (SD)	6.41 ±2.95	2.67 ±1.68	0.74 ±0.41	N/A	N/A	N/A	N/A	0.67 ±0.49	N/A
Smit et al. (2015), INUENDO birth cohort, maternal serum, Greenland and Ukraine 2002–2004 Age 5–9 years	Ukraine (n = 492) Mean	4.88 (2.34–9.94)	0.97 (0.45–2.34)	0.62 (0.30–1.38)	0.16 (0.07–0.35)	N/A	N/A	N/A	1.53 (0.47–4.12)	N/A
	Greenland (n = 532) Mean	20.6 (10.2–49.6)	1.79 (0.80–3.66)	0.73 (0.33–2.01)	0.42 (0.16–1.16)	N/A	N/A	N/A	2.14 (0.97–5.10)	N/A
Timmermann et al. (2017), Children's Health and Environment in the Faroe Islands (CHEF) study, maternal serum, cord blood, and child serum, Faroe Islands 1997–2000 Age 5, 7 and 13	Prenatal (N = 559) Median (IQR)	27.4 (23.3–33.3)	3.3 (2.5–4.0)	0.6 (0.5–0.8)	0.3 (0.2–0.4)	N/A	N/A	N/A	4.5 (2.2–8.3)	N/A
	Age 5/7 (N = 559) Median (IQR)	16.8 (13.5–21.1)	4.0 (3.3–5.0)	1.0 (0.8–1.2)	0.3 (0.2–0.4)	N/A	N/A	N/A	0.6 (0.4–0.9)	N/A
	Age 13 (N = 559) Median (IQR)	6.7 (5.2–8.5)	2.0 (1.6–2.5)	0.7 (0.6; 0.9)	0.3 (0.2–0.4)	N/A	N/A	N/A	0.4 (0.3–0.5)	N/A
Qin et al. (2017), Genetic biomarkers study for Childhood Asthma (GBCA), child serum, Taiwan 2009–2010 Age 10–15 years	w/o asthma Comparison group (n = 168) Median (IQR)	28.83 (12.39–42.02)	0.50 (0.43–0.69)	0.80 (0.62–1.03)	0.93 (0.76–0.93)	N/A	0.48 (0.43–0.52)	N/A	N/A	N/A
	w/asthma GBCA (n = 132) Median (IQR)	31.51 (19.60–91.69)	1.02 (0.48–2.13)	1.00 (0.70–1.25)	1.13 (0.85–1.47)	N/A	0.48 (0.45–0.54)	N/A	N/A	N/A
Zhu et al. (2016), GBCA, child serum, Taiwan 2009–2010 Age 10–15 years	w/o asthma Comparison group (n = 225) Mean ± SD	33.39 ±26.37	1.00 ±1.11	0.87 ±0.34	1.02 ±0.52	N/A	0.48 ±0.20	N/A	2.10 ±2.17	N/A
	w/asthma GBCA (n = 231) Mean ± SD	45.86 ±37.28	1.51 ±1.34	1.07 ±0.47	1.24 ±0.54	N/A	0.53 ±0.20	N/A	3.86 ±8.96	N/A
Zhou et al., 2017a, Zhou et al., 2017b, GBCA, child serum, Taiwan 2009–2010 Age 10–15 years	Girls w/o asthma (n = 123) Median (IQR)	28.8 (14.8–42.6)	0.5 (0.4–1.2)	0.9 (0.6–1.1)	1.0 (0.8–1.2)	N/A	0.5 (0.4–0.5)	3.1 (0.9–6.2)	1.2 (0.5–3.0)	N/A
	Girls w/asthma (n = 73) Median (IQR)	28.2 (13.9–46.0)	0.8 (0.5–1.8)	0.9 (0.7–1.3)	1.1 (0.8–1.5)	N/A	0.5 (0.4–0.6)	2.9 (0.9–7.7)	2.5 (1.3–4.6)	N/A
	Boys w/o asthma (n = 102) Median (IQR)	29.9 (13.0–43.8)	0.5 (0.4–1.4)	0.8 (0.6–1.0)	0.9 (0.8–1.1)	N/A	0.5 (0.4–0.5)	2.4 (0.7–5.9)	1.4 (0.7–2.6)	N/A
	Boys w/asthma (n = 158) Median (IQR)	36.9 (22.6–67.8)	1.3 (0.5–2.3)	1.0 (0.8–1.3)	1.2 (0.9–1.5)	N/A	0.5 (0.5–0.5)	4.3 (1.2–8.5)	2.6 (1.2–4.1)	N/A
Zhou et al., 2017a, Zhou et al., 2017b, GBCA, child serum, Taiwan 2009–2010 Age 10–15 years	w/o asthma (n = 225) Median (IQR)	28.91 (14.06–42.02)	0.52 (0.44–1.27)	0.83 (0.64–1.05)	0.95 (0.76–1.15)	N/A	N/A	N/A	1.32 (0.59–2.79)	N/A
	w/asthma (n = 231) Median (IQR)	33.94 (19.59–61.10)	1.16 (0.48–2.16)	1.00 (0.73–1.28)	1.14 (0.89–1.48)	N/A	N/A	N/A	2.47 (1.25–4.26)	N/A
Humblet et al. (2014), NHANES, child serum, United States 1999–2000 and 2003–2008 Age 12–19 years	Never asthma (n = 1,559) Median (IQR)	16.8 (10.8–26.2)	4.0 (2.8–5.4)	0.8 (0.5–1.2)	N/A	N/A	N/A	N/A	2.0 (1.0–4.1)	N/A
	Ever asthma (n = 318) Median (IQR)	17.0 (10.8–25.8)	4.3 (3.1–5.7)	0.9 (0.6–1.2)	N/A	N/A	N/A	N/A	2.2 (1.1–3.9)	N/A
	No current asthma (n = 1,559) Median (IQR)	16.8 (10.8–26.2)	4.0 (2.8–5.4)	0.8 (0.5–1.2)	N/A	N/A	N/A	N/A	2.0 (1.0–4.1)	N/A
	Current asthma (n = 191) Median (IQR)	16.7 (10.3–25.3)	4.2 (2.9–5.6)	0.9 (0.5–1.3)	N/A	N/A	N/A	N/A	2.1 (1.0–3.9)	N/A
Immune Outcome: Infectious Diseases and Symptoms of Infection										
Dalsager et al., 2016, Odense Child Cohort, maternal serum before 16 weeks gestation, Denmark 2010–2012 Age 3 months, 18 months, and 3 years	All (N = 359) Median (Range)	8.07 (2.36–25.10)	1.68 (0.32–10.12)	0.7 (0.21–3.64)	0.27 (0.10–1.67)	N/A	N/A	N/A	0.32 (0.02–1.03)	N/A
Dalsager et al. (2021), Odense Child Cohort, maternal serum, Denmark 2010–2012 Birth through 4 years	All (N = 1,503) Median (95^th^ percentile)	7.52 (15.08)	1.68 (4.01)	0.64 (1.43)	0.29 (0.79)	N/A	N/A	N/A	0.36 (0.81)	N/A
Goudarzi et al. (2017), Hokkaido Study on Environment and Children's Health, maternal serum at 28–32 weeks gestation, Hokkaido 2003–2008 and 2009 Age 4, 12, and 24 months and 4 years	All (N = 1,558) Mean	5.456	2.713	1.402	0.575	N/A	N/A	N/A	0.322	N/A
Impinen at el., 2018, Environment and Childhood Asthma Cohort, cord blood, Oslo, Norway 1992–1993 Age 0–2 and 10 years	All (N = 641) Mean ± SD	5.6 ±2.3	1.8 ±0.9	0.2 ±0.3	N/A	N/A	N/A	N/A	0.3 ±0.3	N/A
Impinen et el., 2019, Norwegian Mother and Child (MoBa) cohort, maternal plasma, Norway 2003–2004 Age 0–7 years	All (N = 1,943) Mean (IQR)	12.87 (9.92–16.6)	2.54 (1.86–3.30)	0.45 (0.33–0.63)	N/A	N/A	N/A	N/A	0.65 (0.47–0.91)	N/A
Fei et al. (2010), Danish National Birth Cohort, maternal serum at 4–14 weeks gestation, Denmark 1996–2002 Age 0–10.7 years	All (N = 1,400) Mean (Range)	35.3 (6.4–106.7)	5.6 (<LLOQ- 41.5)	N/A	N/A	N/A	N/A	N/A	N/A	N/A
Immune Outcome: Vaccines and Antibodies										
Timmermann et al. (2020). children from Guinea-Bissau, West Africa, Maternal blood sample at inclusion and 9 months and child blood sample at 9 months and 2 years.	135 from the intervention group and 102 from the control group	0.77 (0.53, 1.02)	0.68 (0.53, 0.92)	0.21 (0.13, 0.31)	0.19 (0.15, 0.25)	N/A	N/A	N/A	0.10 (0.09, 0.14)	N/A
Grandjean et al., 2017a, Grandjean et al., 2017b, Faroese Island Cohort 5, child serum, Faroe Islands 2007–2009 Age 18 months and 5 years	Girls, 18 months old (N = 275) Median (IQR)	7.1 (4.5–10.0)	2.8 (2.0–4.5)	1.0 (0.6–1.5)	N/A	N/A	N/A	N/A	0.2 (0.1–0.4)	N/A
	Girls, 5 years old (N = 349) Median (IQR)	4.7 (3.5–6.3)	2.2 (1.8–2.2)	1.1 (0.8–1.6)	N/A	N/A	N/A	N/A	0.3 (0.2–0.4)	N/A
Grandjean et al., 2017a, Grandjean et al., 2017b, Faroe Island Cohort, child serum, Faroe Islands 1997–2000 (birth year) Age 7 and 13 years	7 years old (N = 587) Median (IQR)	15.3 (12.4–19.0)	4.4 (3.5–5.7)	1.1 (0.9–1.5)	0.4 (0.2–0.6)	N/A	N/A	N/A	0.5 (0.4–0.7)	N/A
	13 years old (N = 587) Median (IQR)	6.7 (5.2–8.5)	2.0 (1.6–2.5)	0.7 (0.6–0.9)	0.3 (0.2–0.4)	N/A	N/A	N/A	0.4 (0.3–0.5)	N/A
Grandjean et al. (2012), Faroe Island Cohort, maternal serum at 32 weeks gestation, child serum at age 5, Faroe Islands, 1997–2000 Age 5 and 7 years	Maternal (n = 587) Geometric mean (IQR)	27.3 (23.2–33.1)	3.2 (2.56–4.01)	0.6 (0.46–0.79)	0.28 (0.22–0.38)	N/A	N/A	N/A	4.41 (2.24–8.43)	N/A
	5 years old (n = 537) Geometric mean (IQR)	16.7 (13.5–21.1)	4.06 (3.33–4.96)	1.00 (0.76–1.24)	0.28 (0.21–0.38)	N/A	N/A	N/A	0.63 (0.45–0.88)	N/A
Mogensen et al., 2015b, Mogensen et al., 2015a, Faroe Island Cohort, child serum, Faroe Islands 1997–2000 Age 5 and 7 years	5 years old (N = 459) Median (IQR)	17.3 (14.2–21.3)	4.1 (3.3–5.0)	N/A	N/A	N/A	N/A	N/A	0.6 (5.0–0.9)	N/A
	7 years old (N = 459) Median (IQR)	15.5 (12.8–19.2)	4.4 (3.5–5.7)	N/A	N/A	N/A	N/A	N/A	0.5 (0.4–0.7)	N/A
Pilkerton et al. (2018), NHANES, child serum, U.S. 1999–2004 Age 12–18 years	12–18 years (n = 1, 012) Mean (SE)	25.1 ±0.4	4.8 ±0.7	N/A	N/A	N/A	N/A	N/A	N/A	N/A
Stein et al. (2016), NHANES, child serum, United States 1999–2000, 2003–2003, 2005–2006 Age 12–19 years	12–19 years old 1999–2000 (N = 1,191) Geometric mean	29.0	5.41	0.471	N/A	N/A	N/A	N/A	2.64	N/A
	12–19 years old 2003–2004 (N = 1,191) Geometric mean	19.3	3.89	0.852	N/A	N/A	N/A	N/A	2.44	N/A
Granum et al. (2013)^a^, BraMat, maternal serum at time of delivery, Norway 2007–2008 Age 1–3 years	Maternal (N = 99) Mean (IQR)	5.6 (3.8–7.1)	1.1 (0.8–1.4)	0.3 (0.2–0.4)	N/A	N/A	N/A	N/A	0.3 (0.4–0.3)	N/A
Abraham et al. (2020), Cohort recruited via newspapers and pediatricians, child serum, Germany 1997–1999 Age 1 year	Formula-fed (n = 21) Mean ± SD	6.8 ±3.4	3.8 ±1.1	0.2 ±0.1	N/A	N/A	N/A	N/A	1.7 ±1.1	N/A
	Breastfed (n = 80) Mean ± SD	15.2 ±6.9	16.8 ±6.6	0.6 ±0.2	N/A	N/A	N/A	N/A	2.1 ±1.3	N/A
Zeng et al. (2019), Guangzhou Birth Cohort, cord blood, China 2013 Age 3 months	All (N = 201) Median (IQR)	3.17 (1.88–4.94)	1.22 (0.86–1.74)	0.16 (0.05–0.29)	0.12 (0.05–0.13)	N/A	N/A	N/A	3.96 (2.32–5.41)	10.56 (8.43–15.07)
	Boys (n = 106) Median (IQR)	3.10 (1.76–4.88)	1.23 (0.86–1.94)	0.17 (0.05–0.27)	0.12 (0.05–0.20)	N/A	N/A	N/A	4.15 (2.22–5.76)	10.52 (8.35–14.68)
	Girls (n = 95) Median (IQR)	3.41 (1.95–5.23)	1.21 (0.86–1.66)	0.15 (0.05–0.23)	0.13 (0.05–0.24)	N/A	N/A	N/A	3.74 (2.35–5.12)	10.87 (8.60–15.57)

Note. Acronyms: AD = Atopic dermatitis; GSE = Geometric standard error; IQR = Interquartile range; ku/L = kilo unit per liter; LLOQ = Lower limit of quantitation; ng/mL = Nanogram per milliliter; N/A = Not applicable; SD = Standard deviation, SE = Standard error.

a Pennings et al. (2016) used the same data and methods identified in the Granum et al., (2013) study and thus is not included in the table.

Additional research may elucidate the effect of PFAS on asthma including analyses for various age groups. Gender disparities and changes as children age are frequently reported in asthma research. Prepuberal boys are more likely to have diagnosed asthma and be hospitalized from asthma symptoms, although this difference disappears when entering adolescence (Fuseini and Newcomb, 2017). One study identified a significant positive association between PFOS and asthma only among children ages 3–5 years compared to children ages 10–16 years. The studies included in this review were primarily among individuals older than 10 years of age. Further, preliminary findings may suggest gender plays a role in asthma and PFAS exposure, with two studies reporting stronger associations among males. However, both studies used the same population sample (Genetic and Biomarker Study for Childhood Asthma). Additionally, since prepubescent boys are more likely to have asthma compared to girls of the same age, more research is needed in pre-identified age groups to further understand the potential interaction of PFAS.

A study examining other chemical exposures during the prenatal period and subsequent child wheezing and asthma found preliminary results suggesting a possible association (Hehua et al., 2017). Also, across studies, the parental history of asthma was not frequently adjusted for in the statistical analysis. This is important as research suggests children are three times more likely to have asthma if one parent has diagnosed asthma (Litonjua et al., 1998).

### 4.3 Across the domains

Research on exposure to other environmental agents like dioxins and polychlorinated biphenyls (PCBs) has demonstrated immunodeficiency effects (National Research Council US Subcommittee on Immunotoxicology, 1992). Further, research on twins suggests that immune function is heavily influenced by non-heritable factors. In particular, repeat environmental exposures to herpes viruses, vaccinations, and nutritional factors can result in changes in immune cell frequencies, some of which may outweigh most heritable factors (Brodin et al., 2015). Since immune function can be substantially altered by factors beyond genetics and other persistent organic pollutants have been found to suppress immune function, it is important to understand the possible effect of PFAS exposure on children's immune health.

There are many ubiquitous pollutants that could contribute to environmental exposures for multiple population groups, including women and children. Many of these complex exposures have been shown to also have a cumulative effect on immune function, such as PCBs and air pollutants (Glencross et al., 2020; Kielsen et al., 2016). PFAS studies cited in this review article did not typically ascertain or account for co-exposures to other pollutants nor control for these potential exposures in their assessment of immune functions. The only commonly controlled co-exposure identified was environmental tobacco smoke (see Table 4). Exposures to other pollutants could play a role in the immune function responses seen in these studies, possibly synergistically in conjunction with PFAS exposures. Two studies in our review examined co-exposures between PFAS and PCBs, organochlorine pesticides, phthalate metabolites, and BPA, revealing mixed evidence of immunosuppressive effects (Ashley-Martin et al., 2015; Knudsen et al., 2018). Specific associations were found when examining hematological effects but not for indicators of allergic diseases (i.e., IgE, TSLP and IL-33). Two studies on vaccine response examined the influence of PCB co-exposure due to its immunosuppressive effects. PCB levels only weakly associated with PFAS exposure, and thus co-exposure to PCBs did not significantly alter results (Grandjean et al., 2012; Grandjean et al., 2017a, b). Little is known about these interactions, which could offer further insights into the etiology of immune functions of exposed populations. Additional work is needed to better document both main effects and the role of interaction terms in analytic models.

**Table 4:** Predictors of health outcomes assessed across studies.

Variable^1^	BMI^2^	Breast Feeding	Age	Month of Survey	Daycare Attendance	Exercise	Family History	Parental Education	Nutrition	Race	SES^2^	Sex	Tobacco Exposure	Time since Vaccination	Quantity of Vaccination
Immune Outcome: Allergies															
Ashley-Martin et al. (2015)			X									X			
Averina et al. (2019)	X		X			X		X	X			X	X		
Bamai, 2020	X	X	X				X						X		
Chen et al. (2018)	X	X	X				X				X	X			
Goudarzi et al. (2016)		X	X		X		X	X			X	X	X		
Okada et al. (2012)			X				X				X	X			
Okada et al. (2014)		X	X		X		X	X				X	X		
Oulhote et al. (2017)		X	X									X	X		
Wen et al. (2019)		X	X				X				X	X			
Wen et al. (2019)		X	X				X				X	X			
Wang et al. (2011)		X	X				X					X	X		
Immune Outcome: Asthma															
Agier et al. (2019)	X	X	X					X				X	X		
Beck et al. (2019)	X						X	X				X			
Gaylord et al. (2019)	X		X							X		X	X		
Humblet et al. (2014)			X							X	X	X	X		
Jackson-Browne et al. (2020)			X							X	X	X			
Kvalem et al. (2020)	X		X			X		X							
Manzano-Salgado et al. (2019)	X	X	X					X	X				X		
Smit et al. (2015)		X	X				X	X				X	X		
Timmermann et al. (2017)	X	X			X		X					X	X		
Qin et al. (2017)	X		X	X		X		X				X	X		
Zhu et al. (2016)	X		X	X				X				X	X		
Zhou et al., 2017b, Zhou et al., 2017a	X		X	X		X		X					X		
Zhou et al., 2017b, Zhou et al., 2017a	X		X	X		X		X				X	X		
Immune Outcome: Infectious Diseases and Symptoms of Infection															
Dalsager et al., 2016		X	X		X			X			X	X	X		
Dalsager et al. (2021)		X	X						X		X	X			
Fei et al. (2010)	X	X	X								X	X	X		
Goudarzi et al. (2017)		X	X		X			X				X	X		
Impinen at el., 2018												X			
Impinen et al. (2019)	X		X					X			X		X		
Immune Outcome: Vaccines and Antibodies															
Abraham et al. (2020)														X	X
Dong et al. (2013)	X		X	X				X				X	X		
Grandjean et al. (2012)														X	
Grandjean et al., 2017a, Grandjean et al., 2017b			X									X			
Grandjean et al., 2017a, Grandjean et al., 2017b		X	X									X			
Granum et al. (2013)^c^		X	X				X	X			X	X			
Mogensen et al., 2015a, Mogensen et al., 2015b (DOI 10.1186/s12940-015-0032-9)			X									X			
Pilkerton et al. (2018)	X							X				X			
Stein et al. (2016)	X									X		X			
Timmermann et al., 2020		X									X	X			
Immune Outcome: Other															
Zeng et al. (2019)			X					X			X	X			
Total	18	19	33	5	5	5	12	19	3	4	14	34	21	2	1

Note: Not all confounding variables are included in the chart. Variables were excluded if they were not frequently used across studies (e.g., number of older siblings, time of birth, health insurance, season of conception).

1 Predictor variables included: BMI: As determined for the child, mother, or pre-pregnancy. Breast Feeding: Breast feeding or duration of breast feeding used as a variable, depending on study. Age: Age of the child or mother. Daycare attendance: Whether the child attended daycare. Exercise: Regular exercise (i.e., 1 h per day excluding physical education class.). Family History: Family history of the immune outcome of interest or (e.g., parental asthma, parental atopy). SES: Socioeconomic status of parent(s). Tobacco Exposure: Includes environmental tobacco exposures, secondhand smoke exposures, or smoking.

2 Acronyms: BMI = Body Mass Index; SES = Socioeconomic status.

c Pennings et al. (2016) used the same data and methods identified in the Granum et al., (2013) study and thus is not included in the table.

Further, the studies captured in this review are of background-exposed populations (e.g., dietary intake) rather than areas with moderate to high exposure (see Table 3). It is important to note that data relating to both prenatal and childhood exposure and studies examining optimal susceptibility among children are poorly understood to determine causality. A study investigated whether PFAS exposure adversely affected hematological markers among pregnant women in Greenland (Knudsen et al., 2018). The results showed that decreased lymphocytes, monocytes, neutrophils, platelets, total white blood cell counts, mean corpuscular haemoglobin concentration, and plateletcrit were inversely associated with decreased PFAS serum levels, although further differential risk from specific PFAS was not done (Knudsen et al., 2018). Hematological markers including white blood cells are indicators of immunotoxic outcomes (Tryphonas, 2001) and could have implications for early childhood immune dysfunction. Therefore, research on highly exposed maternal populations such as through occupational studies is warranted. Highly exposed pediatric populations need to be identified. Across studies, the health outcomes had different reporting means such as hospital admissions/medical records, parent reporting, or doctor diagnosis. Studies that use parent reporting are at risk of comprised reliability. For example, Daly et al. (1994) found that parents overreported and underreported episodes of otitis media dependent on chronicity, duration of reporting period, and seriousness of the event. The authors also found increased missing data was found among boys and children with siblings. Similarly, allergic diseases in infants were assessed based on self-administered questionnaires by mother or caretakers raising concern about possible misclassification. Misclassification has the potential to bolster or minimize findings. For example, Goudarzi et al. (2017) describes how, due to the nature of his study, maternal infectious disease reports unverified by medical records may result in “outcome misclassification which generally bias toward the null, or if an association is demonstrated, the true effect may be slightly greater” (p. 137). In addition, postnatal exposure from sources such as food, drinking water or indoor dust was not investigated which may affect exposure assessment.

### 4.4 Limitations

This review had limitations. The search was limited to the PubMed database and therefore articles published in other databases may not have been captured in this review. The search string did not narrow the results by immune-related outcomes. Thus, a large quantity of articles with scoping topics were identified. After the search was conducted, the researchers set the inclusion criteria to only capture immune-related effects for inclusion in the review. We did not further examine the references of relevant articles. The studies included were restricted to English language only. The pool of articles was divided among authors and reviewed for eligibility, interrater reliability between reviewers was not assessed. Lastly, the researchers did not systematically address bias across relevant studies.

## 5 Conclusion

This review summarized effects of PFAS on pediatric immune response including immunosuppression and hypersensitivity outcomes. The immunosuppression findings for vaccine response and infections among children yielded suggestive evidence related to PFAS exposure, particularly PFOS, PFOA and possibly PFHxS and PFNA. Infant and childhood vaccination is intended to offer lasting protection against infectious disease, and antibody level below protection level reflects a deficient immune function. Exposure to PFAS may reduce vaccine efficacy by interference with the immune response. Increased concentration of PFAS in maternal blood was found to be associated with decreased vaccine antibody levels in infants and children. There is preliminary evidence that PFOS exposure is associated with increased risk of infections. However, research across infectious diseases varied significantly with the specific health outcome measured. PFHxS also had preliminary evidence that suggested a possible positive relationship with infection. Future studies should preferentially attempt to quantify immune function impairment from PFAS exposures. Existing evidence for hypersensitivity, allergies, and AD correlation to PFAS exposure was low or inconclusive, suggesting a lack of association for these outcomes.

## Declarations

### Author contribution statement

All authors listed have significantly contributed to the development and the writing of this article.

### Funding statement

This work was supported by Battelle Independent Research & Development Program, USA.

### Data availability statement

Data included in article/supp. material/referenced in article.

### Declaration of interests statement

The authors declare no conflict of interest.

### Additional information

No additional information is available for this paper.

## References

[bib2] Abraham K., Mielke H., Fromme H., Völkel W., Menzel J., Peiser M., Weikert C. (2020). Internal exposure to perfluoroalkyl substances (PFASs) and biological markers in 101 healthy 1-year-old children: associations between levels of perfluorooctanoic acid (PFOA) and vaccine response. Arch. Toxicol..

[bib3] Agency for Toxic Substances and Disease Registry (2018). https://www.atsdr.cdc.gov/toxprofiles/tp200.pdf.

[bib1] Agency for Toxic Substances and Disease Registry (2020). https://www.atsdr.cdc.gov/pfas/health-effects/exposure.html.

[bib4] Agier L., Basagaña X., Maitre L., Granum B., Bird P.K., Casas M., Siroux V. (2019). Early-life exposome and lung function in children in Europe: an analysis of data from the longitudinal, population-based HELIX cohort. Lancet Planet. Health.

[bib5] Ait Bamai Y., Goudarzi H., Araki A., Okada E., Kashino I., Miyashita C., Kishi R. (2020). Effect of prenatal exposure to per- and polyfluoroalkyl substances on childhood allergies and common infectious diseases in children up to age 7 years: the Hokkaido study on environment and children's health. Environ. Int..

[bib6] Ashley-Martin J., Dodds L., Levy A.R., Platt R.W., Marshall J.S., Arbuckle T.E. (2015). Prenatal exposure to phthalates, bisphenol A and perfluoroalkyl substances and cord blood levels of IgE, TSLP and IL-33. Environ. Res..

[bib7] Averina M., Brox J., Huber S., Furberg A.-S., Sørensen M. (2019). Serum perfluoroalkyl substances (PFAS) and risk of asthma and various allergies in adolescents. The Tromsø study Fit Futures in Northern Norway. Environ. Res..

[bib8] Beck I.H., Timmermann C.A.G., Nielsen F., Schoeters G., Jøhnk C., Kyhl H.B., Jensen T.K. (2019). Association between prenatal exposure to perfluoroalkyl substances and asthma in 5-year-old children in the Odense Child Cohort. Environ. Health.

[bib9] Blake B.E., Fenton S.E. (2020). Early life exposure to per- and polyfluoroalkyl substances (PFAS) and latent health outcomes: a review including the placenta as a target tissue and possible driver of peri- and postnatal effects. Toxicology.

[bib10] Blue Cross Blue Shield (2018). https://www.bcbs.com/the-health-of-america/reports/childhood-allergies-america.

[bib11] Bornehag C.G., Nanberg E. (2010). Phthalate exposure and asthma in children. Int. J. Androl..

[bib12] Brodin P., Jojic V., Gao T., Bhattacharya S., Angel Cesar J.L., Furman D., Davis, Mark M. (2015). Variation in the human immune system is largely driven by non-heritable influences. Cell.

[bib13] Cano R.L.E., Lopera H.D.E., Anaya J.M., Shoenfeld Y., Rojas-Villarraga A. (2013). Autoimmunity: from Bench to Bedside.

[bib14] Chandra R.K. (2002). Nutrition and the immune system from birth to old age. Eur. J. Clin. Nutr..

[bib15] Chen Q., Huang R., Hua L., Guo Y., Huang L., Zhao Y., Zhang J. (2018). Prenatal exposure to perfluoroalkyl and polyfluoroalkyl substances and childhood atopic dermatitis: a prospective birth cohort study. Environ. Health.

[bib16] Dalsager L., Christensen N., Halekoh U., Timmermann C.A.G., Nielsen F., Kyhl H.B., Andersen H.R. (2021). Exposure to perfluoroalkyl substances during fetal life and hospitalization for infectious disease in childhood: a study among 1,503 children from the Odense Child Cohort. Environ. Int..

[bib17] Dalsager, Christensen N., Husby S., Kyhl H., Nielsen F., Høst A., Jensen T.K. (2016). Association between prenatal exposure to perfluorinated compounds and symptoms of infections at age 1-4years among 359 children in the Odense Child Cohort. Environ. Int..

[bib18] Daly K.A., Lindgren B., Giebink G.S. (1994). Validity of parental report of a child's medical history in otitis media research. Am. J. Epidemiol..

[bib19] Department of Health and Human Services, Centers for Disease Control and Prevention (2020). https://www.cdc.gov/vaccines/hcp/vis/vis-statements/dtap.html.

[bib20] Dobner J., Kaser S. (2018). Body mass index and the risk of infection - from underweight to obesity. Clin. Microbiol. Infect..

[bib21] Dong G.H., Tung K.Y., Tsai C.H., Liu M.M., Wang D., Liu W., Chen P.C. (2013). Serum polyfluoroalkyl concentrations, asthma outcomes, and immunological markers in a case-control study of Taiwanese children. Environ. Health Perspect..

[bib22] Environmental Protection Agency (2020). https://www.epa.gov/chemical-research/research-and-polyfluoroalkyl-substances-pfas.

[bib23] European Food Safety Authority (2020). Outcome of a public consultation on the draft risk assessment of perfluoroalkyl substances in food. EFSA Support. Publ..

[bib24] Schrenk D., Bignami M., Bodin L., Chipman J.K., Del Mazo J., Schwerdtle T., EFSA Panel on Contaminants in the Food Chain (2020). Risk to human health related to the presence of perfluoroalkyl substances in food. EFSA J. Eur. Food Saf. Author..

[bib25] Fairley K.J., Purdy R., Kearns S., Anderson S.E., Meade B. (2007). Exposure to the immunosuppressant, perfluorooctanoic acid, enhances the murine IgE and airway hyperreactivity response to ovalbumin. Toxicol. Sci..

[bib26] Fei C., McLaughlin J.K., Lipworth L., Olsen J. (2010). Prenatal exposure to PFOA and PFOS and risk of hospitalization for infectious diseases in early childhood. Environ. Res..

[bib27] Fuseini H., Newcomb D.C. (2017). Mechanisms driving gender differences in asthma. Curr. Allergy Asthma Rep..

[bib28] Gaylord A., Berger K.I., Naidu M., Attina T.M., Gilbert J., Koshy T.T., Trasande L. (2019). Serum perfluoroalkyl substances and lung function in adolescents exposed to the World Trade Center disaster. Environ. Res..

[bib29] Glencross D.A., Ho T.R., Camiña N., Hawrylowicz C.M., Pfeffer P.E. (2020). Air pollution and its effects on the immune system. Free Radic. Biol. Med..

[bib30] Goudarzi H., Miyashita C., Okada E., Kashino I., Kobayashi S., Chen C.J., Kishi R. (2016). Effects of prenatal exposure to perfluoroalkyl acids on prevalence of allergic diseases among 4-year-old children. Environ. Int..

[bib31] Goudarzi H., Miyashita C., Okada E., Kashino I., Chen C.J., Ito S., Kishi R. (2017). Prenatal exposure to perfluoroalkyl acids and prevalence of infectious diseases up to 4years of age. Environ. Int..

[bib32] Grandjean P., Andersen E.W., Budtz-Jørgensen E., Nielsen F., Mølbak K., Weihe P., Heilmann C. (2012). Serum vaccine antibody concentrations in children exposed to perfluorinated compounds. JAMA.

[bib33] Grandjean P., Heilmann C., Weihe P., Nielsen F., Mogensen U.B., Budtz-Jørgensen E. (2017). Serum vaccine antibody concentrations in adolescents exposed to perfluorinated compounds. Environ. Health Perspect..

[bib34] Grandjean P., Heilmann C., Weihe P., Nielsen F., Mogensen U.B., Timmermann A., Budtz-Jørgensen E. (2017). Estimated exposures to perfluorinated compounds in infancy predict attenuated vaccine antibody concentrations at age 5-years. J. Immunot..

[bib35] Granum B., Haug L.S., Namork E., Stølevik S.B., Thomsen C., Aaberge I.S., Nygaard U.C. (2013). Pre-natal exposure to perfluoroalkyl substances may be associated with altered vaccine antibody levels and immune-related health outcomes in early childhood. J. Immunot..

[bib36] Greenfeder S., Umland S.P., Cuss F.M., Chapman R.W., Egan R.W. (2001). Th2 cytokines and asthma. The role of interleukin-5 in allergic eosinophilic disease. Respir. Res..

[bib37] Hehua Z., Qing C., Shanyan G., Qijun W., Yuhong Z. (2017). The impact of prenatal exposure to air pollution on childhood wheezing and asthma: a systematic review. Environ. Res..

[bib38] Humblet O., Diaz-Ramirez L.G., Balmes J.R., Pinney S.M., Hiatt R.A. (2014). Perfluoroalkyl chemicals and asthma among children 12-19 years of age: NHANES (1999-2008). Environ. Health Perspect..

[bib39] Impinen A., Nygaard U.C., Lødrup Carlsen K.C., Mowinckel P., Carlsen K.H., Haug L.S., Granum B. (2018). Prenatal exposure to perfluoralkyl substances (PFASs) associated with respiratory tract infections but not allergy- and asthma-related health outcomes in childhood. Environ. Res..

[bib40] Impinen A., Longnecker M.P., Nygaard U.C., London S.J., Ferguson K.K., Haug L.S., Granum B. (2019). Maternal levels of perfluoroalkyl substances (PFASs) during pregnancy and childhood allergy and asthma related outcomes and infections in the Norwegian Mother and Child (MoBa) cohort. Environ. Int..

[bib41] Jackson-Browne M.S., Eliot M., Patti M., Spanier A.J., Braun J.M. (2020). PFAS (per- and polyfluoroalkyl substances) and asthma in young children: NHANES 2013–2014. Int. J. Hyg Environ. Health.

[bib42] Jusko T.A., De Roos Anneclaire J., Lee Sue Y., Thevenet-Morrison K., Schwartz Stephen M., Verner M.-A., Lawrence B.P. (2016). A birth cohort study of maternal and infant serum PCB-153 and DDE concentrations and responses to infant tuberculosis vaccination. Environ. Health Perspect..

[bib43] Kato K., Wong L.Y., Jia L.T., Kuklenyik Z., Calafat A.M. (2011). Trends in exposure to polyfluoroalkyl chemicals in the U.S. Population: 1999-2008. Environ. Sci. Technol..

[bib44] Kato K., Wong L.-Y., Chen A., Dunbar C., Webster G.M., Lanphear B.P., Calafat A.M. (2014). Changes in serum concentrations of maternal poly- and perfluoroalkyl substances over the course of pregnancy and predictors of exposure in a multiethnic cohort of cincinnati, Ohio pregnant women during 2003–2006. Environ. Sci. Technol..

[bib45] Kielsen K., Shamim Z., Ryder L.P., Grandjean P., Heilmann C., E. C. (2016). Environmental Influences on the Immune System.

[bib46] Knudsen A.S., Long M., Pedersen H.S., Bonefeld-Jørgensen E.C. (2018). Persistent organic pollutants and haematological markers in Greenlandic pregnant women: the ACCEPT sub-study. Int. J. Circumpolar Health.

[bib47] Kvalem H.E., Nygaard U.C., Lødrup Carlsen K.C., Carlsen K.H., Haug L.S., Granum B. (2020). Perfluoroalkyl substances, airways infections, allergy and asthma related health outcomes - implications of gender, exposure period and study design. Environ. Int..

[bib48] Le Deist, Fischer (2008).

[bib49] Litonjua A.A., Carey V.J., Burge H.A., Weiss S.T., Gold D.R. (1998). Parental history and the risk for childhood asthma. Does mother confer more risk than father?. Am. J. Respir. Crit. Care Med..

[bib50] Lloyd C.M., Hessel E.M. (2010). Functions of T cells in asthma: more than just TH2 cells. Nat. Rev. Immunol..

[bib51] MacGillivray D.M., Kollmann T.R. (2014). The role of environmental factors in modulating immune responses in early life. Front. Immunol..

[bib52] Manzano-Salgado C.B., Granum B., Lopez-Espinosa M.-J., Ballester F., Iñiguez C., Gascón M., Casas M. (2019). Prenatal exposure to perfluoroalkyl substances, immune-related outcomes, and lung function in children from a Spanish birth cohort study. Int. J. Hyg Environ. Health.

[bib53] Mogensen, Grandjean P., Heilmann C., Nielsen F., Weihe P., Budtz-Jørgensen E. (2015). Structural equation modeling of immunotoxicity associated with exposure to perfluorinated alkylates. Environ. Health.

[bib54] Mogensen, Grandjean P., Nielsen F., Weihe P., Budtz-Jørgensen E. (2015). Breastfeeding as an exposure pathway for perfluorinated alkylates. Environ. Sci. Technol..

[bib55] Muñoz-Carrillo J.L., Castro-García F.P., Chávez-Rubalcaba F., Chávez-Rubalcaba I., Martínez- Rodríguez J.L., Hernández-Ruiz M.E., Athari S.S. (2017). Immunoregulatory Aspects of Immunotherapy.

[bib56] National Heart Lung, Blood Institute (2013). https://www.nhlbi.nih.gov/health/educational/wecan/downloads/energy-worksheet.pdf.

[bib57] National Research Council US Subcommittee on Immunotoxicology (1992).

[bib58] Nielsen N.M., Hansen A.V., Simonsen J., Hviid A. (2011). Prenatal stress and risk of infectious diseases in offspring. Am. J. Epidemiol..

[bib59] Okada E., Sasaki S., Saijo Y., Washino N., Miyashita C., Kobayashi S., Kishi R. (2012). Prenatal exposure to perfluorinated chemicals and relationship with allergies and infectious diseases in infants. Environ. Res..

[bib60] Okada E., Sasaki S., Kashino I., Matsuura H., Miyashita C., Kobayashi S., Kishi R. (2014). Prenatal exposure to perfluoroalkyl acids and allergic diseases in early childhood. Environ. Int..

[bib61] Oulhote Y., Shamim Z., Kielsen K., Weihe P., Grandjean P., Ryder L.P., Heilmann C. (2017). Children's white blood cell counts in relation to developmental exposures to methylmercury and persistent organic pollutants. Reprod. Toxicol..

[bib62] Pachkowski B., Post G.B., Stern A.H. (2019). The derivation of a Reference Dose (RfD) for perfluorooctane sulfonate (PFOS) based on immune suppression. Environ. Res..

[bib63] Pai U.A., Chandrasekhar P., Carvalho R.S., Kumar S. (2018). The role of nutrition in immunity in infants and toddlers: an expert panel opinion. Clin. Epidemiol. Glob. Health.

[bib64] Palmer A.C. (2011). Nutritionally mediated programming of the developing immune system. Adv. Nutr..

[bib65] Pennings J.L., Jennen D.G., Nygaard U.C., Namork E., Haug L.S., van Loveren H., Granum B. (2016). Cord blood gene expression supports that prenatal exposure to perfluoroalkyl substances causes depressed immune functionality in early childhood. J. Immunot..

[bib66] Pilkerton C.S., Hobbs G.R., Lilly C., Knox S.S. (2018). Rubella immunity and serum perfluoroalkyl substances: sex and analytic strategy. PLoS One.

[bib67] Qin X.D., Qian Z.M., Dharmage S.C., Perret J., Geiger S.D., Rigdon S.E., Dong G.H. (2017). Association of perfluoroalkyl substances exposure with impaired lung function in children. Environ. Res..

[bib68] Rappazzo K.M., Coffman E., Hines E.P. (2017). Exposure to perfluorinated alkyl substances and health outcomes in children: a systematic review of the epidemiologic literature. Int. J. Environ. Res. Publ. Health.

[bib69] Rivera Rivera N.Y., Tamayo-Ortiz M., Mercado García A., Just A.C., Kloog I., Téllez-Rojo M.M., Rosa M.J. (2020). Prenatal and early life exposure to particulate matter, environmental tobacco smoke and respiratory symptoms in Mexican children. Environ. Res..

[bib70] Scepanovic P., Alanio C., Hammer C., Hodel F., Bergstedt J., Patin E., for The Milieu Intérieur, C (2018). Human genetic variants and age are the strongest predictors of humoral immune responses to common pathogens and vaccines. Genome Med..

[bib71] Smit L.A., Lenters V., Høyer B.B., Lindh C.H., Pedersen H.S., Liermontova I., Heederik D. (2015). Prenatal exposure to environmental chemical contaminants and asthma and eczema in school-age children. Allergy.

[bib72] Smith T., Cunningham-Rundles C. (2019). Primary B-cell immunodeficiencies. Hum. Immunol..

[bib73] Somanunt S., Chinratanapisit S., Pacharn P., Visitsunthorn N., Jirapongsananuruk O. (2017). The natural history of atopic dermatitis and its association with Atopic March. Asian Pac. J. Allergy Immunol..

[bib74] Stein C.R., McGovern K.J., Pajak A.M., Maglione P.J., Wolff M.S. (2016). Perfluoroalkyl and polyfluoroalkyl substances and indicators of immune function in children aged 12-19 y: National Health and Nutrition Examination Survey. Pediatr. Res..

[bib75] Steinke J.W., Borish L. (2001). Th2 cytokines and asthma. Interleukin-4: its role in the pathogenesis of asthma, and targeting it for asthma treatment with interleukin-4 receptor antagonists. Respir. Res..

[bib76] Timmermann, Budtz-Jørgensen E., Jensen T.K., Osuna C.E., Petersen M.S., Steuerwald U., Grandjean P. (2017). Association between perfluoroalkyl substance exposure and asthma and allergic disease in children as modified by MMR vaccination. J. Immunot..

[bib77] Timmermann, Jensen Kristoffer J., Nielsen F., Budtz-Jørgensen E., van der Klis F., Benn Christine S., Fiskerane B. (2020). serum perfluoroalkyl substances, vaccine responses, and morbidity in a cohort of Guinea-bissau children. Environ. Health Perspect..

[bib78] Tryphonas H. (2001). Approaches to detecting immunotoxic effects of environmental contaminants in humans. Environ. Health Perspect..

[bib79] Wan X.C., Woodruff P.G. (2016). Biomarkers in severe asthma. Immunol. Allergy Clin..

[bib80] Wang I.J., Hsieh W.-S., Chen C.-Y., Fletcher T., Lien G.-W., Chiang H.-L., Chen P.-C. (2011). The effect of prenatal perfluorinated chemicals exposures on pediatric atopy. Environ. Res..

[bib81] Wen H.J., Wang S.L., Chen P.C., Guo Y.L. (2019). Prenatal perfluorooctanoic acid exposure and glutathione s-transferase T1/M1 genotypes and their association with atopic dermatitis at 2 years of age. PLoS One.

[bib82] Wen H.J., Wang S.L., Chuang Y.C., Chen P.C., Guo Y.L. (2019). Prenatal perfluorooctanoic acid exposure is associated with early onset atopic dermatitis in 5-year-old children. Chemosphere.

[bib83] Winkens K., Vestergren R., Berger U., Cousins I.T. (2017). Early life exposure to per- and polyfluoroalkyl substances (PFASs): a critical review. Emerg. Contam..

[bib84] Xue J., Zartarian V., Moya J., Freeman N., Beamer P., Black K., Shalat S. (2007). A meta-analysis of children's hand-to-mouth frequency data for estimating nondietary ingestion exposure. Risk Anal..

[bib85] Zeng X.W., Bloom M.S., Dharmage S.C., Lodge C.J., Chen D., Li S., Dong G.H. (2019). Prenatal exposure to perfluoroalkyl substances is associated with lower hand, foot and mouth disease viruses antibody response in infancy: findings from the Guangzhou Birth Cohort Study. Sci. Total Environ..

[bib86] Zhou, Hu L.-W., Qian Z., Chang J.-J., King C., Paul G., Dong G.-H. (2016). Association of perfluoroalkyl substances exposure with reproductive hormone levels in adolescents: by sex status. Environ. Int..

[bib87] Zhou, Bao W.W., Qian Z.M., Dee Geiger S., Parrish K.L., Yang B.Y., Dong G.H. (2017). Perfluoroalkyl substance exposure and urine CC16 levels among asthmatics: a case-control study of children. Environ. Res..

[bib88] Zhou, Hu L.W., Qian Z.M., Geiger S.D., Parrish K.L., Dharmage S.C., Dong G.H. (2017). Interaction effects of polyfluoroalkyl substances and sex steroid hormones on asthma among children. Sci. Rep..

[bib89] Zhu, Qin X.D., Zeng X.W., Paul G., Morawska L., Su M.W., Dong G.H. (2016). Associations of serum perfluoroalkyl acid levels with T-helper cell-specific cytokines in children: by gender and asthma status. Sci. Total Environ..

